# Small language models applied in text summarization task of health-related news to improve public health audit: an experimental case study

**DOI:** 10.3389/frai.2026.1708993

**Published:** 2026-02-05

**Authors:** Alysson Guimarães, Methanias Colaço Junior, Samuel Santana De Almeida, Gabriely Garcia Ferreira de Araújo, Raphael Silva Fontes, Helder Prado, Luca Pareja Credidio Freire Alves, Natan Matos, Ricardo Alexsandro de Medeiros Valentim, João Paulo Queiroz dos Santos

**Affiliations:** 1 Postgraduate Program in Computer Science (PROCC), Federal University of Sergipe, São Cristóvão, Brazil; 2 Laboratory for Technological Innovation in Health (LAIS), Onofre Lopes University Hospital, Federal University of Rio Grande do Norte (UFRN), Natal, Brazil; 3 Department of Science and Technology, Federal University of Rio Grande do Norte, Natal, Brazil; 4 Center for Innovation and Advanced Technology (NAVI), Federal Institute of Rio Grande do Norte (IFRN), Natal, Brazil

**Keywords:** text summarization, automatic text summarization, abstractive generic summarization, extractive generic summarization, small language models

## Abstract

**Context:**

Fraud and corruption are among the main crimes affecting public institutions, with the healthcare sector being particularly vulnerable due to its structural complexity, the coexistence of public and private providers, the large number of actors involved, the globalized nature of supply chains, the high financial costs, and the information asymmetry among stakeholders. These factors weaken healthcare systems, resulting in resource waste, reduced resilience during medical emergencies, and limited access to essential services.

**Objective:**

This study aims to evaluate automatic text summarization methods by comparing the quality of machine-generated summaries with those produced by humans, from the perspective of Data Scientists and SUS Auditors, within the context of audits carried out by the National Department of Unified Health System (Sistema Único de Saúde—SUS) Auditing (AudSUS).

**Method:**

A controlled experiment was conducted to assess the performance of Small Language Models (SLMs) in summarization tasks, using the metrics ROUGE-N, ROUGE-L, BLEU, METEOR, and BERTScore. In addition, the consistency of results across 35 runs, their contribution to reducing information overload, and their pairwise performances were evaluated.

**Results:**

The models NousResearch/Hermes-3-Llama-3.2-3B, Qwen/Qwen2.5-7B-Instruct, and meta-llama/Llama-3.2-3B-Instruct achieved the highest average performances across all metrics, standing out for their ability to preserve contextual meaning and synthesize essential information more effectively than human-generated summaries.

**Conclusion:**

The findings highlight the potential of SLMs as tools to reduce information overload, thereby enhancing the effectiveness of the analytical phase of audits and enabling faster preparation of teams for the operational stage.

## 1 Introduction

Currently, most of the user-generated data in society is unstructured and textual, and Natural Language Processing (NLP) has gradually been applied in public administration. Governments and public institutions have employed this technology to process large volumes of documents with the aim of improving the quality of public services, increasing citizens' trust in institutions, and enhancing efficiency and effectiveness, particularly in functional areas such as healthcare, education, and decision-making (Jiang et al., 2023).

Many governments need to analyze, in real time, multiple sources of information, both static and dynamic, to monitor public and private cameras, citizens' comments on social media, online transactions, and events. This analysis seeks to identify patterns, establish correlations, and build predictive models that enable strategy optimization and improvement of services offered to citizens. Another key objective is to ensure the monitoring and surveillance necessary to protect the population and mitigate the impact of crimes (Benjelloun et al., 2015).

Among these crimes, fraud and corruption stand out, with the healthcare sector being particularly vulnerable due to several factors. These include the complexity of health systems, which combine public and private providers; the large number of people involved; the globalized nature of supply chains; high public and private expenditures; and information asymmetry among actors, which can negatively affect decision-making in the sector. Such vulnerabilities weaken healthcare systems, resulting in resource waste, reduced resilience during medical emergencies, and compromised coverage and access to essential health services (Mackey et al., 2018).

A study conducted by Transparency International, a global civil society organization against corruption, revealed that more than 50% of citizens in 42 out of 109 surveyed countries, believe that the healthcare sector in their country is corrupt or highly corrupt. Furthermore, the World Health Organization (WHO) estimated that, out of the US$ 7.5 trillion spent on healthcare worldwide in 2008, US$ 415 billion (7.3%) was lost due to fraud or corruption in the sector (Mackey et al., 2018).

The impacts of corruption extend beyond financial losses, encompassing social consequences, particularly in low-income countries. In these regions, both immediate and long-term effects include increased morbidity and mortality, due to the barriers created by corruption in accessing healthcare services, disproportionately affecting the most vulnerable groups. Corruption undermines the quality of healthcare systems and distorts the allocation of investments in the sector (Mackey et al., 2018).

Fraud also damages organizational reputation and public trust, making the implementation of strategies to prevent, detect, and mitigate these risks vital. One effective mechanism in combating fraud is the use of ombudsman offices and reporting channels, which play a central role in compliance systems by enabling the receipt and handling of fraud and corruption complaints (Paula et al., 2024).

Furthermore, audits represent another crucial tool to mitigate these crimes and their impacts. However, the large volume of data presents significant challenges, including information overload, which makes the auditing process complex and difficult to conduct (do Amaral et al., 2020; Paula et al., 2024).

The auditing process is generally costly, time-consuming, and requires substantial human and material resources. Therefore, it is necessary to implement solutions and techniques that automate the analysis of corruption reports. This process is typically divided into two stages: in the first, the goal is to identify elements and evidence of corruption, such as suppliers, contracts, employees, clients, and other stakeholders, by assessing the plausibility and consistency of the reports and fraud indicators; in the second stage, the investigation itself takes place (Paula et al., 2024).

To build the knowledge required for audit work, information gathering about the audit's objectives must be carried out. At this stage, various sources are used, including websites (Fontes et al., 2023), and, to support the information collection process, web scraping techniques for large-scale data extraction from health-related websites can be employed. For analyzing this large volume of data, NLP techniques such as text summarization can be applied, reducing the time and resources required for the analysis and evidence collection of potential irregularities (Sanchez-Gomez et al., 2022; Benjelloun et al., 2015; Madureira et al., 2021).

Based on this scenario, this article evaluates and proposes the use of small language models, following the experimental process described in **?** and **?**, in order to investigate the ability of these models to support the auditing process and to identify the most suitable models for this task. The main motivation is to support the auditing process by reducing information overload in the analytical phase of planning.

The article is structured as follows: Section 2 presents the literature on small language models. Section 3 details the dataset employed, the process of creating the reference set, and the evaluation metrics adopted. In Section 4, the experiment's objectives, planning, research questions, dependent and independent variables, selection of study objects, experimental design, and instrumentation are specified. Section 5 describes the procedures for data preparation, execution, and validation. Subsequently, Section 6 presents the results obtained and discusses the threats to validity. Finally, Section 7 provides the concluding remarks and proposes directions for future work.

## 2 Related work

Large Language Models (LLMs) have driven a paradigm shift in Natural Language Processing (NLP) (**?**). These models have demonstrated emergent capabilities in text generation, question answering, and reasoning, facilitating tasks across multiple domains (Wang et al., 2025). The field of NLP has been profoundly transformed by the ability of LLMs to perform downstream tasks after being trained on massive datasets under a self-supervised learning regime (**?**).

Transformer-based models, such as BERT, RoBERTa, mT5, and the GPT family, have become the foundation for a wide range of applications and research directions in NLP (**?**). The applications of LLMs in NLP encompass a wide range of tasks, including Question Answering (QA) (Ren, 2024), text classification or categorization into predefined classes (e.g., sentiment, fake news, topics) (Fields et al., 2024), text summarization (Van Veen et al., 2024), sentiment analysis or polarity detection (positive, negative, or neutral) (Ren, 2024), and Named Entity Recognition (NER) (Luo et al., 2023). These diverse tasks are employed across domains such as health, education, and industry (Raza et al., 2025).

The development of LLMs has rapidly expanded, encompassing both proprietary models such as ChatGPT, Bard, and Claude, and open-source models such as Llama (Wang et al., 2025). Current research largely focuses on model scalability, training data, efficiency, and the overall capabilities of these architectures (**?**).

As argued in **?**, despite these advancements, progress with LLMs has not occurred uniformly across languages. Most models are trained on high-resource languages, such as English, whereas multilingual models typically exhibit lower performance than their monolingual counterparts. This disparity arises from imbalances in training corpora, in which high-resource languages predominate, ultimately resulting in user dissatisfaction with multilingual models when applied to low-resource languages.

The practical use of LLMs still faces several limitations, including high computational costs and restrictive licensing regimes, privacy and data security concerns, infeasibility on low-power or edge devices, high inference latency, and suboptimal performance in specialized domains (Wang et al., 2025; **?**; Corrêa et al., 2025). An alternative to mitigate these constraints lies in the adoption of Small Language Models (SLMs).

Small Language Models (SLMs) have gained increasing attention as promising alternatives to LLMs. The exact definition of SLMs may vary; however, they are generally characterized by having fewer parameters than LLMs, with some classifications considering models with fewer than one billion parameters. Other definitions classify them as “small” relative to their larger counterparts, encompassing models with up to 10 billion parameters, while emphasizing the absence of emergent abilities typically observed in larger LLMs (Wang et al., 2025).

SLMs stand out for their low inference latency, cost-effectiveness, development efficiency, and ease of customization and adaptability (Wang et al., 2025). They offer significant computational savings in both pre-training and inference, requiring reduced memory and storage, which is particularly relevant for applications with constrained resources (Wang et al., 2025). Such characteristics make SLMs especially suitable for resource-limited environments, including edge devices and real-time applications, where they can enhance privacy, security, and response times through on-device processing (Wang et al., 2025; **?**). Moreover, when fine-tuned for specific domains, SLMs can match or even surpass the performance of larger models in specialized tasks (Wang et al., 2025).

Text summarization can be either abstractive or extractive. An abstractive summary generates new content that does not exist in the original document, creating novel words and sentences. In contrast, an extractive summary selects a subset of sentences from the original text to form the summary (Sanchez-Gomez et al., 2020). Furthermore, summaries can be classified as generic or query-oriented. Generic summaries do not require any user input (Sanchez-Gomez et al., 2018), whereas query-oriented summaries require some form of user-provided information, typically a query or topic of interest in sentence form (Sanchez-Gomez et al., 2024). Additionally, summarization methods can be categorized as single-document or multi-document. Single-document methods condense the information from a single text into a concise summary, while multi-document methods extract key information from a set of documents (Saini et al., 2019). Summarization approaches can also be classified as supervised or unsupervised (Alguliyev et al., 2015).

This study focuses on the application of SLMs for the NLP task of automatic text summarization. For this purpose, the following models were employed: BART (Lewis et al., 2019), Gemma (Gemma Team et al., 2024), Sabiá (Pires et al., 2023), Llama (Team, 2024a), TeenyTinyLlama (**?**), Hermes (Teknium et al., 2024), Qwen (Team, 2024b), and Tucano (Corrêa et al., 2025). Additionally, extractive models were considered, including TextRank (Nenkova and McKeown, 2011), LexRank (Erkan and Radev, 2004), LSA (Steinberger and JeŽek, 2004), KLSum (Haghighi and Vanderwende, 2009), and SumBasic (Woodsend and Lapata, 2011).

## 3 Materials and methods

This is an experimental study, following the steps outlined by **?** for evaluating the results of text summarization methods applied to health-related news articles with indications of irregularities, assessing the quality of summaries using the ROUGE-N, ROUGE-L, BLEU, METEOR, and BERTScore metrics.

The description of the dataset employed, the process of selecting news articles with evidence of irregularities, and the evaluation metrics for the automatic summaries are presented in Sections 3.1–3.3, respectively.

The replication of experiments is a key characteristic of any scientific field. In the software domain, it is therefore essential to apply methods that can be replicated and evaluated, in order to prevent new methods, techniques, languages, and tools from being proposed, published, or marketed without proper experimentation and validation (Travassos et al., 2020).

### 3.1 Database collection

The construction of the database, including all stages of data collection and storage, lies outside the scope of this study. This Section describes the process conducted by (Fontes et al. 2023), which was carried out in three stages. Table 1 describes the stages of database collection. The first stage consisted of a proof of concept based on interviews with auditors from National Department of Unified Health System (Sistema Único de Saúde—SUS) Auditing (AudSUS), aiming to clarify the process of acquiring the material used in the analytical phase of auditing. The audit process implemented by AudSUS consists of a set of activities and the preparation of documents that specify the tasks to be performed. These tasks are organized into three phases: the analytical phase, the operational (in loco) phase, and the final report. The analytical phase corresponds to audit planning, whose purpose is to prepare the team for the operational stage. This preparation involves building the necessary knowledge for the audit through the collection of information related to its objectives. The operational phase comprises the audit itself, while the final report presents the audit's overall findings.

**Table 1:** Database creation stages.

**Stage**	**Summary**
1. Proof of concept	Conducted interviews with auditors from the National Department of SUS Auditing (AudSUS) to clarify how the materials used in the analytical auditing phase are obtained.
2. Exploratory study	Mapped how textual news articles are organized in the sources, examining publication routines, periodicity, limitations, and solutions for missing or incomplete data. This stage resulted in the initial design of a data storage model.
3. Database construction	Developed the full data collection pipeline using Python, Django, PostgreSQL, and OpenSearch. Implemented specialized crawlers to gather and update news articles across multiple sources with varying publication frequency. The final database contains over 6 million articles selected based on auditor recommendations.
4. Preprocessing pipeline	Performed text cleaning and metadata standardization to ensure data quality and proper storage in both the database and search index.

The second stage involved an exploratory study aimed at mapping how textual news articles are organized in the sources, identifying their publication routines, periodicity, existing limitations, and possible solutions for cases of missing or incomplete data. Upon completion of this mapping, the construction of a model responsible for storing this data was initiated.

In the third stage, the database itself was built. For this purpose, a data collection pipeline was developed using the Python programming language, the Django framework, the PostgreSQL database, and OpenSearch for indexing the results. The collection of news articles was performed with specialized crawlers capable of reading, interpreting, and collecting metadata from the sources. These crawlers were configured to collect only new articles or to reprocess previous publications. This functionality enables more frequent searches in sources that publish several articles daily, as opposed to those that publish only once a day, monthly, or occasionally, such as audit reports and official journals. The final database contains more than 6 million textual articles from research sources indicated by auditors consulted in the initial stage.

After collection, the articles undergo a preprocessing pipeline designed to clean the texts and standardize their metadata according to the established model. This step is essential to ensure data quality and its proper storage in both the database and the indexing tool.

### 3.2 Health-related news selection

The information collected by (Fontes et al. 2023) was organized into a database containing 154,407 news articles with metadata regarding publication date, source (website), news title, headline, and full content. All data manipulation processes were conducted using Jupyter Notebook,^1^ with the Polars library^2^ version 1.32.0, and the Python programming language^3^ version 3.10.12.

To identify health-related news articles with potential indications of audit relevance, a three-stage keyword-based selection strategy was applied. In the first stage, across the entire dataset, articles containing the keyword “saúde” (health) in their content (corpus) were classified as “Health” while all others were classified as “Generic News.” The generic articles were excluded from subsequent stages.

In the second stage, keywords indicating potential irregularities were applied to the subset of articles previously classified as “Health,” dividing them into “Generic Health" and “Health with Irregularities.” Articles containing at least one of the keywords in the title and/or headline were classified as “Irregularity News." At this stage, 6,239 articles were identified as containing indications of irregularity, while the remaining 3,277 were classified as “Generic Health.” Table 2 presents the list of keywords used.

**Table 2:** Keywords used to identify signs of irregularities.

**Keywords**
abuso, abuso de poder, acordo ilegal, acusaç, acusação, apropriação indébita, auditoria, aumento orçamento, bilhões, cartel, coação, compra, compras públicas, conluio, contrato, contratos, corrupto, corrupção, corte orçamento, crime, crime organizado, criminoso, deflagrou, denuncia, denúncia, desassistência, desfalque, desonestidade, desonesto, desperdício, desvio, desvios, disfarce, documento alterado, dolo, enganar, engano, enganos, enganoso, enriquecimento, enriquecimento ilícito, escândalo, esquema, evasão, falcatrua, falsa declaração, falsificado, falsificador, falsificação, falso, falta, falta de equipamentos, falta equipamento, fiscalização, forjar, fraudador, fraudar, fraude, fraude em contratos, fraude em licitações, fraude financeira, fraude licitação, fraudulento, fugir, golpe, ilegal, ilusão, ilícito, indicativo, indício, infração, investiga, investigação, irregular, irregularidade, irregularidade administrativa, irregularidade de gestão, irregularidade financeira, irregularidades, lavagem, lavagem de dinheiro, licitação, mandado, manipulado, manipulador, manipulação, manipulação de dados, maquiar, mentira, milhões, má conduta, negligência dolosa, ocultar, ocultação, PF, peculato, perjúrio, plano, plano de saúde, plano saúde, prevaricação, propina, recurso, relatório falso, rombo, roubo, sem autorização, sem consentimento, sobrefaturamento, sonegar, sonegação, suborno, sugestão, superfaturamento, suspeit, suspeita, suspeito, transação, transação suspeita, transgressão, transparência, uso indevido, uso indevido de recursos, plano saude, uso irregular, venda.

In the third stage, articles belonging to the “Irregularity News” subgroup were independently assessed by two annotators. A substantial inter-rater agreement (IRA) (Landis and Koch, 1977) was achieved, with a Cohen's Kappa (*k*) value of 0.6203. Table 3 presents the contingency table of the evaluations. Five additional evaluators were assigned to resolve cases of disagreement in the classification. Each evaluator was individually responsible for making a final decision on 65 distinct samples. The assessment involved reviewing the titles and abstracts of all articles to confirm their categorization as “Health Irregularity,” “Generic Health,” or “Generic News.”

**Table 3:** Contingency matrix of annotations between Evaluator 1 and Evaluator 2.

**Evaluator 1 \Evaluator 2**	**Generic news**	**Health (Generic)**	**Health (irregularity)**	**Total Linha (*R*_*i*_)**
Generic news	4,532	302	145	4,979
Health (generic)	146	676	7	829
Health (irregularity)	211	17	203	431
**Total (C_*i*_)**	**4,889**	**995**	**355**	***N* = 6, 239**

**Figure 1:**
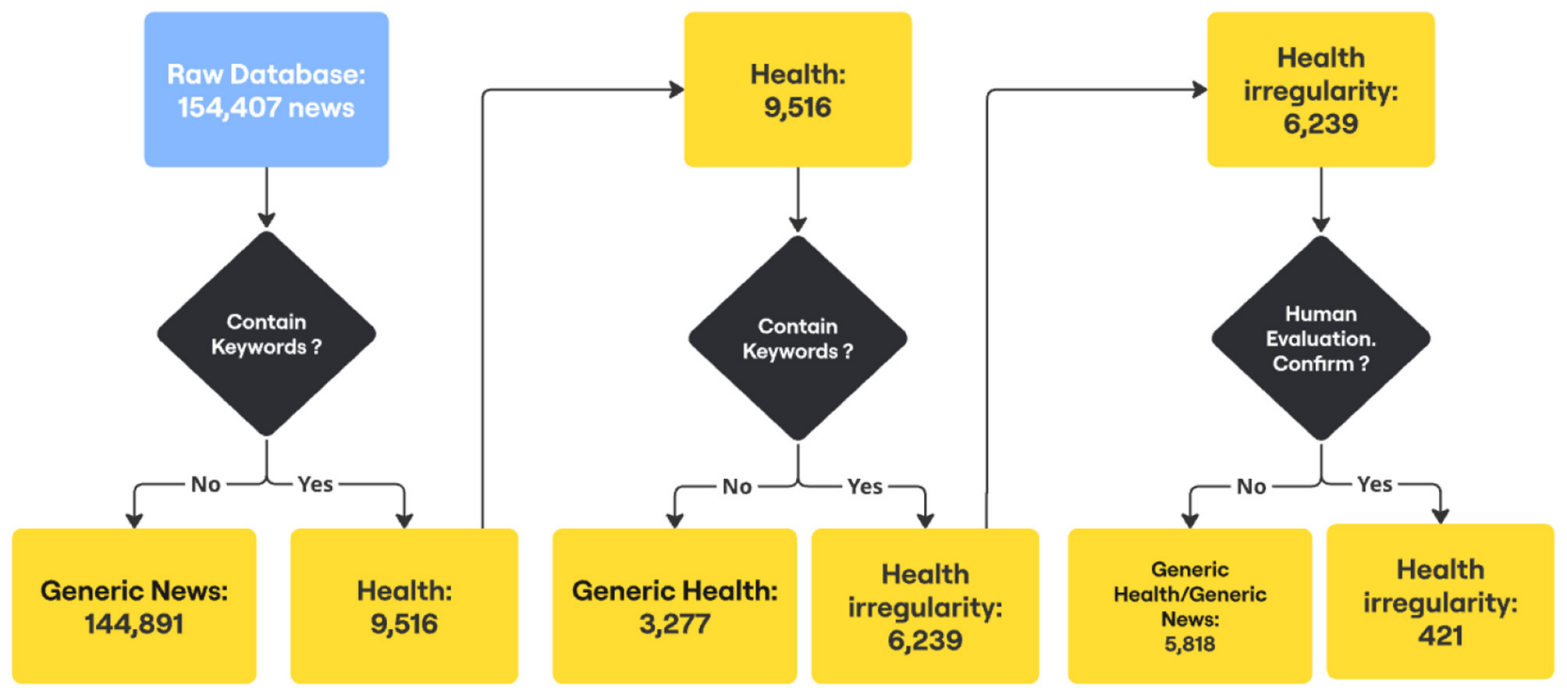
Health-related news selection process.

Finally, after the third stage, 421 health-related articles with indications of irregularity were identified. Figure 1 illustrates the news selection process.

For the use of this dataset in the text summarization task, it was necessary to create summaries of the original articles to serve as reference summaries for the evaluation of the automatically generated ones. These reference summaries were produced by an external journalist researcher.

### 3.3 Evaluation metrics

This subsection describes the evaluation metrics adopted in the experiment. The metrics employed were ROUGE-N (Lin, 2004), ROUGE-L (Lin, 2004), BLEU (Papineni et al., 2002), METEOR (Lavie and Agarwal, 2007), and BERTScore (Zhang et al., 2020).

#### 3.3.1 ROUGE-N

The Recall-Oriented Understudy for Gisting Evaluation (ROUGE-N) (Lin, 2004) is widely employed in the literature to assess the quality of summaries generated by automatic summarization methods. This metric evaluates summary quality by measuring the overlap of word sequence units (N-grams) and word pairs between the automatically generated summary and the reference summary (El-Kassas et al., 2021). The formal definition is given in Equation 1.


$$\begin{array}{l} \text{ROUGE-N}=\frac{\sum_{S\in \text{RefSummaries}}\sum_{\text{N-grams}\in S}{\text{Count}}_{\text{match}}\left(\text{N-gram}\right)}{\sum_{S\in \text{RefSummaries}}\sum_{\text{N-grams}\in S}\text{Count}\left(\text{N-gram}\right)} \end{array}$$ (1)

where *RefSummaries* denotes the set of reference summaries used for comparison with the automatically generated summary, *N*-grams refer to consecutive segments of *N* words (or *tokens*) in a sentence or text, Count_match_(N-gram) represents the number of times a specific *N*-gram from the reference summary appears in the generated summary—thus indicating the count of overlapping *N*-grams between the reference and generated summaries—while Count(N-gram) is the total count of *N*-grams in the reference summary. The denominator's summation accounts for all possible *N*-grams that could have been captured from the reference summary.

#### 3.3.2 ROUGE-L

The Recall-Oriented Understudy for Gisting Evaluation—Longest Common Subsequence (ROUGE-L) (Lin, 2004) is an automatic evaluation measure for text quality, based on the computation of the Longest Common Subsequence (LCS) between a reference text *R* and an automatically generated text *H*.

Let *LCS*(*R, H*) denote the length of the longest common subsequence between *R* and *H*. Recall, Precision, and Fβ (F1-score) are defined by Equations 2–4, respectively. Here, |*R*| represents the length of the reference sequence and |*H*| the length of the generated sequence. For the computation of Fβ, the parameter β is typically set to 1, yielding the F1-score.

The use of the longest common subsequence provides ROUGE-L with the ability to capture the global structure and the relative order of words, without the strict contiguity constraints required by N-grams. This characteristic distinguishes ROUGE-L from ROUGE-N, allowing it to reflect textual similarity at a more flexible level.


$$\begin{array}{l} \text{R\_LCS}=\frac{LCS\left(R,H\right)}{\left|R\right|} \end{array}$$ (2)

$$\begin{array}{l} \text{P\_LCS}=\frac{LCS\left(R,H\right)}{\left|H\right|} \end{array}$$ (3)

$$\begin{array}{l} \text{F\_LCS}=\frac{\left(1+\beta^{2}\right)\cdot R\text{\_}LCS\cdot P\text{\_}LCS}{R\text{\_}LCS+\beta^{2}\cdot P\text{\_}LCS} \end{array}$$ (4)

#### 3.3.3 BLEU

The Bilingual Evaluation Understudy (BLEU) metric (Papineni et al., 2002) measures the quality of a generated text based on the precision of N-grams with respect to one or more references, while incorporating a brevity penalty to avoid excessively short summaries. Formally, let *p*_*n* denote the *n*-gram precision of order *n*, with weights *w*_*n* (typically uniform), and *BP* the brevity penalty. where |*H*| is the length of the generated summary and |*R*| is the length of the reference summary. It is defined as follows:


$$\begin{array}{l} \text{BLEU}=BP\cdot \exp\left(\overset{N}{\underset{n=1}{\sum }}w_{n}\log p_{n}\right) \end{array}$$ (5)

$$BP=\{\begin{array}{ll} 1 & \text{if }|H|>|R|\\ e^{\left(1-|R|/|H|\right)} & \text{if }|H|\leq |R| \end{array}$$

#### 3.3.4 METEOR

The Metric for Evaluation of Translation with Explicit ORdering (METEOR) (Lavie and Agarwal, 2007) establishes a flexible alignment between automatically generated summaries and references, accounting for exact matches, stems, synonyms, and paraphrases. Precision *P* and recall *R* are defined over the identified matches. The F_α score is computed as shown in Equation 6, where α balances the relative importance of precision and recall. A fragmentation penalty *Pen*, dependent on the dispersion of matched segments, is also applied. Finally, METEOR is computed according to Equation 7.


$$\begin{array}{l} F_{\alpha }=\frac{PR}{\alpha P+\left(1-\alpha \right)R} \end{array}$$ (6)

$$\begin{array}{l} \text{METEOR}=\left(1-Pen\right)\cdot F_{\alpha } \end{array}$$ (7)

#### 3.3.5 BERTScore

The BERTScore metric (Zhang et al., 2020) is grounded in semantic representations obtained from pretrained Transformer-based language models. Given a set of embeddings *e*_*h* for tokens in the automatically generated summary and *e*_*r* for tokens in the reference, where cos(*e*_*h, e*_*r*) denotes the cosine similarity between embeddings, BERTScore diverges from traditional metrics by capturing actual semantics, including synonyms and paraphrases. Moreover, it correlates strongly with human evaluation, since both the reference text and the generated summary are represented contextually through embeddings. BERTScore is computed as shown in Equation 10.


$$\begin{array}{l} P=\frac{1}{\left|H\right|}\underset{h\in H}{\sum }\underset{r\in R}{\max}\cos\left(e_{h},e_{r}\right) \end{array}$$ (8)

$$\begin{array}{l} R=\frac{1}{\left|R\right|}\underset{r\in R}{\sum }\underset{h\in H}{\max}\cos\left(e_{r},e_{h}\right) \end{array}$$ (9)

$$\begin{array}{l} \text{F\_BERT}=\frac{2PR}{P+R} \end{array}$$ (10)

## 4 Experimental definition

This section presents the objective of the experimental evaluation, the planning, the research questions, the independent and dependent variables, and the hypotheses.

### 4.1 Objective

To formalize the objective of this study, the Goal Question Metric (GQM) model proposed by (Basili and Weiss 1984) was employed. The aim of this work is to analyze automatic text summarization methods, with the purpose of evaluating the quality of automatically generated summaries, against human-generated summaries, with respect to metrics of ROUGE-1, ROUGE-2, ROUGE-L, BLEU, METEOR and BERTScore, from the perspective of Data Scientists and Auditors of the Brazilian Unified Health System (SUS), in the context of public health audits conducted by the National Department of Unified Health System (Sistema Único de Saúde—SUS) Auditing (AudSUS).

### 4.2 Planning

The experiment was conducted in a controlled environment, using text summarization methods on health-related news articles with indications of irregularities. Table 4 presents and describes the methods employed.

**Table 4:** Models used, their characteristics and purposes.

**Model name**	**Task**	**Language**	**Sum. type**	**Type**
Google/gemma-2b-it	Text generation	English	Abstractive	Fine-tuned
KLSum	N/A	Multilanguage	Extractive	Graph-based
LexRank	N/A	Multilanguage	Extractive	Graph-based
LSA	N/A	Multilanguage	Extractive	Math/Statistics
Maritaca-ai/sabia-7b	Text generation	Portuguese (BR)	Abstractive	Pre-trained
Meta-llama/Llama-3.2-3B-Instruct	Text generation	Multilanguage	Abstractive	Fine-tuned
Nicholaskluge/TeenyTinyLlama-460m-Chat	Text generation	Portuguese (BR)	Abstractive	Pre-trained
NousResearch/Hermes-3-Llama-3.2-3B	Text generation	English	Abstractive	Fine-tuned
Qwen/Qwen2.5-0.5B-Instruct	Text generation	English	Abstractive	Fine-tuned
Qwen/Qwen2.5-1.5B-Instruct	Text generation	English	Abstractive	Fine-tuned
Qwen/Qwen2.5-7B-Instruct	Text generation	English	Abstractive	Fine-tuned
SumBAsic	N/A	Multilíngue	Extractive	Math/Statistics
TextRank	N/A	Multilíngue	Extractive	Graph-based
TucanoBR/Tucano-1b1-Instruct	Text Generation	Portuguese (BR)	Abstractive	Fine-tuned
TucanoBR/Tucano-2b4-Instruct	Text Generation	Portuguese (BR)	Abstractive	Fine-tuned

Sorted alphabetically by Model Name.

The experiment involved the generation and evaluation of automatic summaries, as well as the analysis and presentation of results.

The automatic summary generation phase consisted of applying text summarization methods to the database of news articles with indications of irregularities. The database employed is described in Section 3.1.

For the evaluation, quality measurement metrics for summaries were applied, including ROUGE-N (Lin, 2004), ROUGE-L (Lin, 2004), BLEU (Papineni et al., 2002), METEOR (Lavie and Agarwal, 2007), and BERTScore (Zhang et al., 2020). These metrics are detailed in Section 5.3.

In the subsequent phase, analyses were conducted on the mean scores of each applied method, and hypothesis testing was performed to evaluate differences in results. To determine whether there were statistically significant differences in mean performance among the methods, a paired non-parametric Wilcoxon Signed-Rank test was applied.

After identifying the best-performing methods, they were comparatively described regarding the distribution of results and internal consistency through descriptive analysis of the standard deviation of the evaluation metrics. Finally, threats to validity are presented.

### 4.3 Context selection

Despite technological advancements, many processes in the public sector still rely on manual searches for knowledge construction. This scenario is also observed in the AudSUS, responsible for the control and oversight of the Brazilian Unified Health System (SUS). Audit activities conducted by this department play a crucial role in the management and proper use of public resources; however, the process is highly resource-intensive due to the high demand, as auditors must oversee all SUS areas while also addressing internal and external demands from the Ministry of Health (Fontes et al., 2023). In this context, the proposed experiment aims to support the analytical phase of the audit process, which is responsible for planning and preparing the team for the operative phase, through the collection of information related to the audit objectives.

### 4.4 Research question

To guide the experiment and fulfill the objectives of this study, the following research questions were formulated:

- RQ1: Can an automatic text summarization method support the audit process by reducing information overload regarding indications of irregularities?
- RQ2: Among the selected summarization methods, which are the top three in terms of summary quality?

To address the research questions, the following theoretical hypotheses, presented in Table 5, were formulated.

**Table 5:** Research questions and associated hypotheses.

**RQ**	**Null hypothesis (*H*_0)**	**Alternative hypothesis (*H*_1)**
RQ1	Automatic summarization methods do not match human performance.	Automatic summarization methods match human performance.
RQ2	The evaluated automatic summarization methods do not show statistically significant differences.	The evaluated automatic summarization methods show statistically significant differences.

### 4.5 Dependent variables

The dependent variables, or output variables, were the automatic summaries generated by the models, from which the summary quality evaluation metrics ROUGE-N, ROUGE-L, BLEU, METEOR, and BERTScore were derived.

### 4.6 Independent variables

In this experiment, the independent variable, or input variable, is the reference dataset created for evaluating the automatic summaries, as well as the tested models: abstractive models BART (Lewis et al., 2019), Gemma (Gemma Team et al., 2024), Sabiá (Pires et al., 2023), Llama (Team, 2024a), TeenyTinyLlama (**?**), Hermes (Teknium et al., 2024), Qwen (Team, 2024b), and Tucano (Corrêa et al., 2025), and extractive models TextRank (Nenkova and McKeown, 2011), LexRank (Erkan and Radev, 2004), LSA (Steinberger and JeŽek, 2004), KLSum (Haghighi and Vanderwende, 2009), and SumBasic (Woodsend and Lapata, 2011). These models were selected following the classification in (Wang et al. 2025), i.e., models with up to 10 billion parameters.

### 4.7 Objects selection

Following the context described in Section 4.3, the objects of this experiment are health-related news articles with indications of irregularities, as described in Section 3.2. The dataset contains 421 news articles and human-generated reference summaries.

To generalize the results of this experiment to the broader population of news articles, it is necessary to evaluate the results using a representative sample (**?**). For sample size calculation, a finite population of 154,407 news articles (the total number of articles in the complete dataset) was considered. It is noteworthy that the final sample exceeds the estimated number according to Equation 12. For sample size calculation, a 95% confidence level (*Z* = 1.96), a tolerable sampling error of 5% (*e* = 0.05), and an expected proportion of 50% (*p* = 0.5) were used, maximizing sample variability and ensuring a more conservative sample size.

The sample size calculation for a finite population was conducted in two steps: first, the sample for an infinite population (*n*) was estimated using Equation 11, and then the adjustment for a finite population (*n*_*adjusted*_) was applied according to Equation 12, resulting in approximately 383.21 samples, as shown in Equation 14.


$$\begin{array}{l} n=\frac{Z^{2}\cdot p\cdot \left(1-p\right)}{e^{2}} \end{array}$$ (11)

$$\begin{array}{l} n_{ajustado}=\frac{n}{1+\left(\frac{n-1}{N}\right)} \end{array}$$ (12)

$$\begin{array}{l} n=\frac{1,96^{2}\cdot 0,5\cdot \left(1-0,5\right)}{0,05^{2}}=\frac{3,8416\cdot 0,25}{0,0025}=384,16 \end{array}$$ (13)

$$\begin{array}{l} n_{ajustado}=\frac{384,16}{1+\left(\frac{384,16-1}{154407}\right)}\approx 383,21 \end{array}$$ (14)

### 4.8 Experiment design

Automatic summaries were generated in 35 independent rounds for each of the 421 news articles, resulting in 14,735 automatic summaries per method. A total of 35 rounds were conducted to ensure that the distribution of the sample mean score for each method approaches a normal distribution, even if the underlying population distribution does not follow one, in accordance with the Central Limit Theorem (CLT). This sample size (*n* = 35) surpasses the commonly accepted threshold of *n*≥30 for the application of the CLT, thereby enabling the subsequent use of robust parametric tests (which assume normality of the sampling distribution) and ensuring a minimum number of observations for normality tests, such as the Kolmogorov–Smirnov test (**?**).

In this experiment, the metrics ROUGE-N (Lin, 2004), ROUGE-L (Lin, 2004), BLEU (Papineni et al., 2002), METEOR (Lavie and Agarwal, 2007), and BERTScore (Zhang et al., 2020) were employed to measure the quality of the generated summaries, as described in Section 3.3. These metrics were applied to evaluate the automatic summaries produced by the methods, using human-generated summaries as references.

For extractive methods, a preprocessing step was required, in which words were normalized using stemming, a process that reduces words to their root forms, decreasing linguistic variation and complexity while preserving the essential meaning. This process is necessary to ensure consistent representation of variant forms of the same word by removing suffixes, thereby achieving textual normalization (**?**).

The maximum length of the automatic summaries was constrained to the average length of the reference summaries. For extractive methods, summaries were limited to a maximum of five sentences, corresponding to the average sentence count of the reference summaries (4.92 sentences). For abstractive summaries, reference summaries were tokenized, the mean number of tokens was calculated, and a minimum summary length was set as *max*(5, *mean*_*tokens*−*tolerance*_*value*), while the maximum length was fixed at *mean*_*tokens*+*tolerance*_*value*. The tolerance value was defined as (*mean*_tokens*0.1). Tokenization, which divides text into subword units called tokens, was automated using the tokenizer specific to the method being executed. The restriction on summary length aims to ensure a fairer comparison, as recommended in NIST (2025).

### 4.9 Instrumentation

The following materials and resources were employed:

- Google Sheets;
- Annotated database with reference summaries (Section 3.1);
- Google Colab^4^;
- Python programming language (3.11.13)^5^;
- Python libraries: accelerate (1.9.0), bert-score (0.3.12), bitsandbytes (0.47.0), datasets (4.0.0), evaluate (0.4.5), matplotlib (3.10.3), openpyxl (3.1.4), packaging (25.0), pandas (2.2.3), polars (1.32.0), protobuf (6.31.1), pyarrow (20.0.0), python-dotenv (1.1.1), rouge-score (0.1.2), seaborn (0.13.2), sentencepiece (0.1.99), sumy (0.11.0), tiktoken (0.9.0), tqdm (4.67.1), scipy (1.15.3), and transformers (4.54.0);
- Computational resources from the High-Performance Computing Center (NPAD) at the Federal University of Rio Grande do Norte (UFRN).

## 5 Experiment operation

This section describes the preparation of the experiment, its execution, and the evaluation of the results.

### 5.1 Experiment preparation

The database containing all news articles was obtained as described in Section 3.1. The reference summaries, used as the comparison standard, were produced by an independent researcher, ensuring that for each health-related news article with indications of irregularity, a corresponding summary was created.

Before applying the summarization methods, a virtual environment was set up for dependency management, ensuring reproducibility and compatibility across different development environments. Within this environment, all required libraries for method execution were installed.

In the case of automatic summaries generated by extractive methods, a preprocessing step was carried out, in which the news texts were normalized using stemming, thereby reducing linguistic variation and complexity while preserving the essential meaning of words.

To ensure systematic execution of the process, a summarization pipeline was developed. This pipeline consists of a script capable of receiving one or more methods and repeatedly generating automatic summaries, with the number of repetitions (*N*) defined as 35 in this experiment.

As a pilot study, the pipeline was initially tested in five rounds with 25 samples to verify its functionality. Necessary adjustments and corrections were made during this preliminary stage (**?**). Subsequently, the complete process was executed for all methods. Table 6 presents the hyperparameters employed for the abstractive models.

**Table 6:** Hyperparameters abstractive models.

**Hyperparameter**	**Value**
Truncation	True
Padding	“longest”
Return_tensors	“pt”
Do_sample	True
Top_k	100
Top_p	0.95
Temperature	1.0
Num_return_sequences	1

### 5.2 Experiment execution

The execution of the experiment consisted of two main stages: the generation and evaluation of the automatic summaries.

In the generation stage, the pipeline was executed for each method using the database of news articles with indications of fraud.

The generation stage involved the implementation of a pipeline in Python, automating the execution of each method for 35 iterations. The pipeline for extractive methods is presented in Algorithm 1, while the pipeline for abstractive methods is shown in Algorithm 2. The materials and resources used are detailed in Section 4.9.

Algorithm 1 Extractive text summarization pipeline.

1: *df*[news_content_prep]                                ←    preprocessing(*df*[news_content])
2: *n*_*round*←35
3: *dfs*_*por*_*round*←[ ]
4: *apply*_*rounds*←True
5: **if** *apply*_*rounds* = True **then**
6:    **for** *round*←1 **to** *n*_*round* **do**
7:        **for** *summarizer*∈*summarizers* **do**
8:           *df*_*round*←copy(*df*)
9:           *df*_*round*[summary]                     ←               *summarizer*(*df*_*round*[news_content_prep])
10:           *df*_*round*[method]←*summarizer*
11:           *df*_*round*[round]←*rodada*
12:           append(*dfs*_*por*_*round, df*_*round*) 
13:        **end for** 
14:      **end for**
15:      *df*_*final*←concat(*dfs*_*por*_*round*) 
16: **else**
17:    *df*_*final*←save_results 
18: **end if**

Algorithm 2 Abstractive text summarization pipeline.

1: *n*_*round*←35
2: **for** *model*_*name*∈*models*_*to*_*use* **do**
3:      *model*_*args*←generate_args(*model*_*name*)
4:      *save*_*path*←create_output_folder(*model*_*name*)
5:      **for** *round*←1 **to** *n*_*round* **do**
6:         *df*_*with*_*sum*←*dataset*.map(summarize(*model*_*args*))
7:         write_parquet(*df*_*round*, *out*_*file*) 
8:      **end for**
9:      free_memory(*model*, *tokenizer*) 
10: **end for**

**Figure 2:**
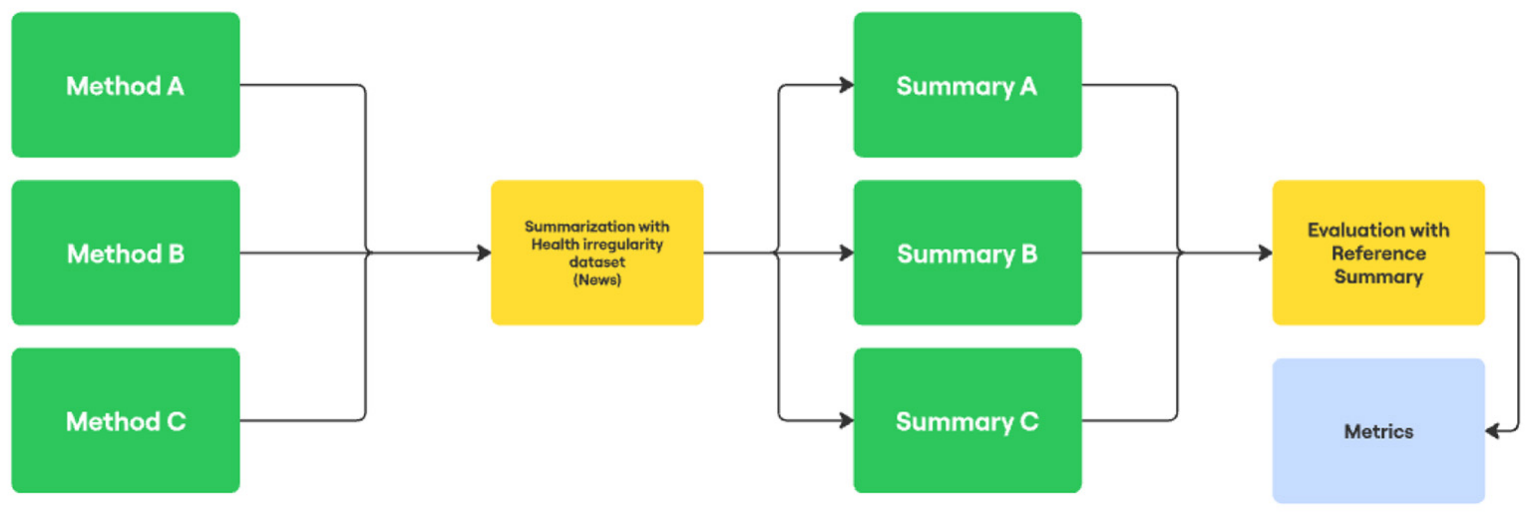
Text summarization process and evaluation.

After the generation of summaries, the evaluation stage was initiated, consisting of measuring the quality of the automatic summaries using the ROUGE-N (Lin, 2004), ROUGE-L (Lin, 2004), BLEU (Papineni et al., 2002), METEOR (Lavie and Agarwal, 2007), and BERTScore (Zhang et al., 2020) metrics, with the reference summaries serving as the ground truth. Each of the 14,735 summaries generated by method was compared with its corresponding manually produced reference summary. Figure 2 illustrates the execution process.

### 5.3 Data evaluation

Five (5) types of statistical tests were applied for analysis, interpretation, and validation: Anderson–Darling (AD Test), Kolmogorov–Smirnov (KS Test), Wilcoxon Signed-Rank Test (pairwise), *Z*-score, and Interquartile Range (IQR). The Anderson–Darling (AD Test) and Kolmogorov–Smirnov (KS Test) were employed to assess data normality, while the Wilcoxon Signed-Rank Test (pairwise) was applied to compare the medians of the ROUGE-1, ROUGE-2, ROUGE-L, BLEU, METEOR, and BERTScore metrics. *Z*-score and Interquartile Range (IQR) metrics were used to identify outliers within the evaluation metrics.

## 6 Results

This section presents the process of data analysis and interpretation, threats to validity, and conclusions and future work.

### 6.1 Data analysis and interpretation

**Figure 3:**
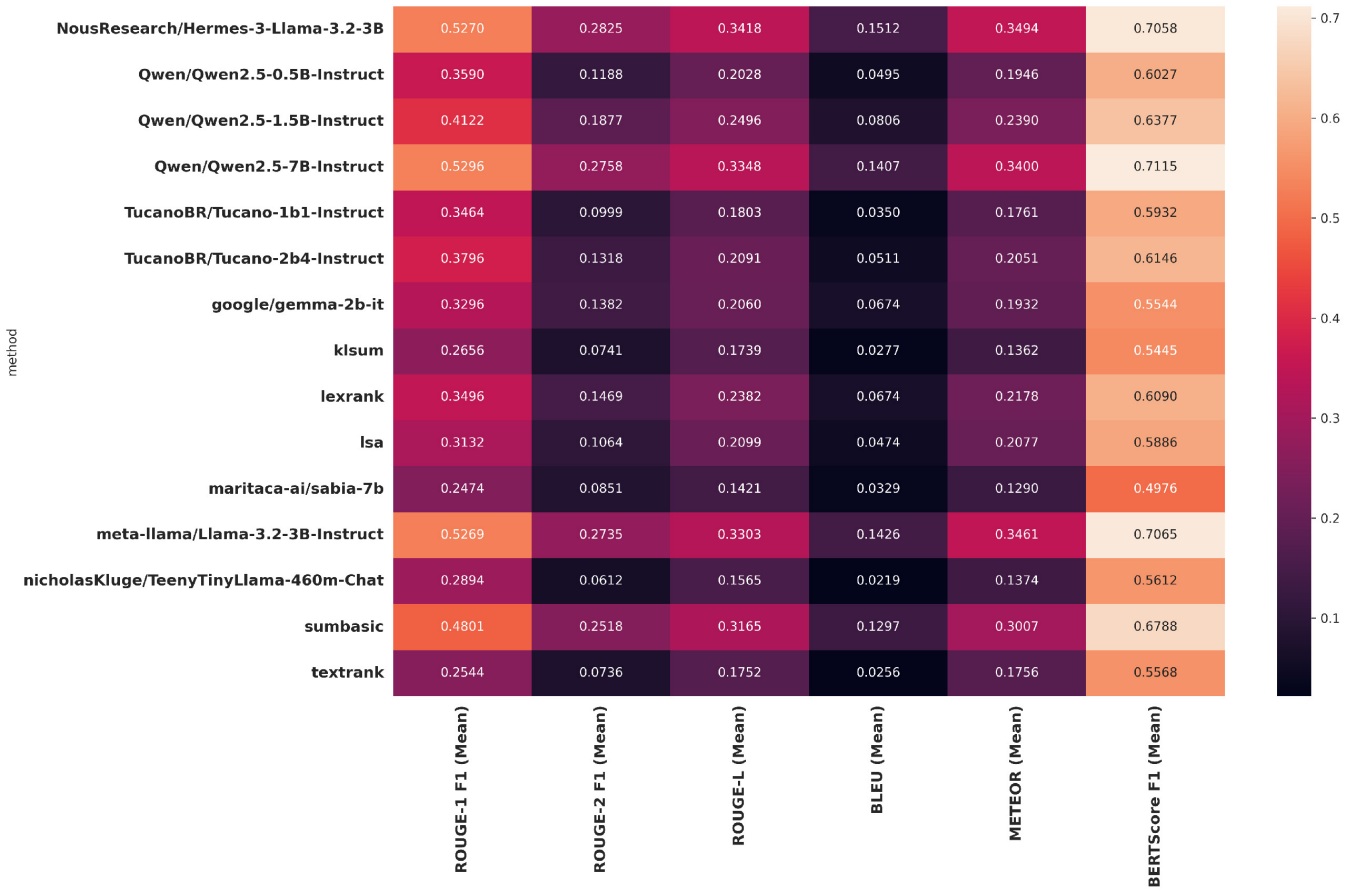
Heatmap of evaluation metrics results.

To address the research questions outlined in Section 4.4, the Execution stage was followed, and the results for the evaluation metrics were obtained. Table 7 presents the results of the ROUGE-1 (Table 7), ROUGE-2 (Table 7), ROUGE-L (Table 7), BLEU (Table 7), METEOR (Table 7), and BERTScore (Table 7) metrics, aggregated by mean, minimum value, maximum value, and standard deviation for each metric. Figure 3 provides a visual representation of the metrics in the form of a heatmap, which the lighter the color, the higher the score.

**Table 7:** Results for the evaluation metrics.

**Method**	**Mean**	**Median**	**Min**	**Max**	**Std**
**Results for the ROUGE-1 F1 metric**					
Qwen/Qwen2.5-7B-Instruct	0.5296	0.5310	0.2135	0.7638	0.0860
NousResearch/Hermes-3-Llama-3.2-3B	0.5270	0.5350	0.1618	0.7873	0.0930
Meta-llama/Llama-3.2-3B-Instruct	0.5269	0.5309	0.2198	0.7279	0.0833
Sumbasic	0.4801	0.4892	0.1597	0.7451	0.1018
Qwen/Qwen2.5-1.5B-Instruct	0.4122	0.4494	0.0000	0.7372	0.1547
TucanoBR/Tucano-2b4-Instruct	0.3796	0.3950	0.0000	0.6792	0.1079
Qwen/Qwen2.5-0.5B-Instruct	0.3590	0.3588	0.0000	0.6783	0.0992
lexrank	0.3496	0.3459	0.0333	0.7644	0.1243
TucanoBR/Tucano-1b1-Instruct	0.3464	0.3475	0.0000	0.6460	0.0961
Google/gemma-2b-it	0.3296	0.3952	0.0000	0.7244	0.2063
Lsa	0.3132	0.3089	0.0559	0.6636	0.1011
Nicholaskluge/TeenyTinyLlama-460m	0.2894	0.2971	0.0000	0.6196	0.0951
Klsum	0.2656	0.2624	0.0000	0.5796	0.1099
Textrank	0.2544	0.2541	0.0245	0.5539	0.0882
Maritaca-ai/sabia-7b	0.2474	0.2538	0.0000	0.6797	0.1650
**Results for the ROUGE-2 F1 metric**					
NousResearch/Hermes-3-Llama-3.2-3B	0.2825	0.2835	0.0177	0.6411	0.0972
Qwen/Qwen2.5-7B-Instruct	0.2758	0.2709	0.0143	0.6429	0.0932
Meta-llama/Llama-3.2-3B-Instruct	0.2735	0.2678	0.0287	0.5809	0.0880
Sumbasic	0.2518	0.2500	0.0135	0.5743	0.1049
Qwen/Qwen2.5-1.5B-Instruct	0.1877	0.1897	0.0000	0.5745	0.1024
Lexrank	0.1469	0.1278	0.0000	0.5830	0.1035
Google/gemma-2b-it	0.1382	0.1319	0.0000	0.5317	0.1268
TucanoBR/Tucano-2b4-Instruct	0.1318	0.1258	0.0000	0.5000	0.0776
Qwen/Qwen2.5-0.5B-Instruct	0.1188	0.1073	0.0000	0.4842	0.0770
Lsa	0.1064	0.0926	0.0000	0.4569	0.0808
TucanoBR/Tucano-1b1-Instruct	0.0999	0.0902	0.0000	0.4351	0.0662
Maritaca-ai/sabia-7b	0.0851	0.0610	0.0000	0.5118	0.0909
Klsum	0.0741	0.0574	0.0000	0.3704	0.0696
Textrank	0.0736	0.0639	0.0000	0.3448	0.0533
Nicholaskluge/TeenyTinyLlama-460m	0.0612	0.0478	0.0000	0.4136	0.0521
**Results for the ROUGE-L metric**					
NousResearch/Hermes-3-Llama-3.2-3B	0.3418	0.3376	0.1036	0.6990	0.0938
Qwen/Qwen2.5-7B-Instruct	0.3348	0.3246	0.1268	0.6533	0.0886
Meta-llama/Llama-3.2-3B-Instruct	0.3303	0.3187	0.1423	0.6754	0.0860
Sumbasic	0.3165	0.3136	0.0881	0.6209	0.1001
Qwen/Qwen2.5-1.5B-Instruct	0.2496	0.2551	0.0000	0.6423	0.1050
Lexrank	0.2382	0.2261	0.0333	0.5571	0.0903
Lsa	0.2099	0.1977	0.0466	0.5522	0.0708
TucanoBR/Tucano-2b4-Instruct	0.2091	0.2042	0.0000	0.5370	0.0705
Google/gemma-2b-it	0.2060	0.2239	0.0000	0.5950	0.1307
Qwen/Qwen2.5-0.5B-Instruct	0.2028	0.1916	0.0000	0.5550	0.0661
TucanoBR/Tucano-1b1-Instruct	0.1803	0.1729	0.0000	0.4733	0.0549
Textrank	0.1752	0.1704	0.0228	0.4412	0.0566
Klsum	0.1739	0.1705	0.0000	0.4706	0.0733
Nicholaskluge/TeenyTinyLlama-460m	0.1565	0.1550	0.0000	0.4167	0.0497
Maritaca-ai/sabia-7b	0.1421	0.1433	0.0000	0.5564	0.0979
**Results for the BLEU metric**					
NousResearch/Hermes-3-Llama-3.2-3B	0.1512	0.1475	0.0053	0.4962	0.0796
Meta-llama/Llama-3.2-3B-Instruct	0.1426	0.1344	0.0056	0.4693	0.0733
Qwen/Qwen2.5-7B-Instruct	0.1407	0.1351	0.0059	0.4522	0.0766
Sumbasic	0.1297	0.1266	0.0015	0.3923	0.0801
Qwen/Qwen2.5-1.5B-Instruct	0.0806	0.0685	0.0000	0.4298	0.0661
Google/gemma-2b-it	0.0674	0.0404	0.0000	0.4446	0.0765
Lexrank	0.0674	0.0445	0.0001	0.4253	0.0666
TucanoBR/Tucano-2b4-Instruct	0.0511	0.0379	0.0000	0.3600	0.0447
Qwen/Qwen2.5-0.5B-Instruct	0.0495	0.0337	0.0000	0.3694	0.0470
Lsa	0.0474	0.0309	0.0022	0.3533	0.0481
TucanoBR/Tucano-1b1-Instruct	0.0350	0.0212	0.0000	0.3087	0.0375
Maritaca-ai/sabia-7b	0.0329	0.0134	0.0000	0.3511	0.0510
Klsum	0.0277	0.0124	0.0000	0.3187	0.0393
Textrank	0.0256	0.0147	0.0011	0.1964	0.0285
Nicholaskluge/TeenyTinyLlama-460m	0.0219	0.0120	0.0000	0.2662	0.0276
**Results for the METEOR metric**					
NousResearch/Hermes-3-Llama-3.2-3B	0.3494	0.3448	0.0793	0.6754	0.1007
Meta-llama/Llama-3.2-3B-Instruct	0.3461	0.3366	0.0985	0.6782	0.0963
Qwen/Qwen2.5-7B-Instruct	0.3400	0.3312	0.0980	0.6957	0.0972
Sumbasic	0.3007	0.2908	0.0400	0.6983	0.1148
Qwen/Qwen2.5-1.5B-Instruct	0.2390	0.2420	0.0000	0.6947	0.1169
Lexrank	0.2178	0.2042	0.0125	0.6407	0.1124
Lsa	0.2077	0.1938	0.0520	0.5133	0.0879
TucanoBR/Tucano-2b4-Instruct	0.2051	0.2008	0.0000	0.5700	0.0843
Qwen/Qwen2.5-0.5B-Instruct	0.1946	0.1813	0.0000	0.6275	0.0834
Google/gemma-2b-it	0.1932	0.2100	0.0000	0.6630	0.1486
TucanoBR/Tucano-1b1-Instruct	0.1761	0.1663	0.0000	0.5314	0.0701
Textrank	0.1756	0.1637	0.0323	0.5115	0.0747
Nicholaskluge/TeenyTinyLlama-460m	0.1374	0.1338	0.0000	0.5027	0.0630
Klsum	0.1362	0.1247	0.0000	0.5361	0.0854
Maritaca-ai/sabia-7b	0.1290	0.1095	0.0000	0.6382	0.1088
**Results for the BERTScore F1 metric**					
Qwen/Qwen2.5-7B-Instruct	0.7115	0.7114	0.5404	0.8646	0.0525
Meta-llama/Llama-3.2-3B-Instruct	0.7065	0.7089	0.5330	0.8548	0.0482
NousResearch/Hermes-3-Llama-3.2-3B	0.7058	0.7102	0.5086	0.8505	0.0511
Sumbasic	0.6788	0.6848	0.4802	0.8205	0.0582
Qwen/Qwen2.5-1.5B-Instruct	0.6377	0.6470	0.4184	0.8793	0.0704
TucanoBR/Tucano-2b4-Instruct	0.6146	0.6248	0.2273	0.7964	0.0783
lexrank	0.6090	0.6074	0.3028	0.8276	0.0761
Qwen/Qwen2.5-0.5B-Instruct	0.6027	0.6019	0.3337	0.8031	0.0633
TucanoBR/Tucano-1b1-Instruct	0.5932	0.5963	0.2574	0.7688	0.0612
Lsa	0.5886	0.5917	0.4159	0.7730	0.0646
Nicholaskluge/TeenyTinyLlama-460m	0.5612	0.5685	0.2039	0.7445	0.0647
Textrank	0.5568	0.5552	0.4025	0.7337	0.0536
Google/gemma-2b-it	0.5544	0.6254	0.1784	0.8563	0.1752
Klsum	0.5445	0.5522	0.1199	0.7367	0.0847
Maritaca-ai/sabia-7b	0.4976	0.5178	0.2273	0.7901	0.1257

Given these results, it is observed that the abstractive models led performance across almost all metrics with consistent results, demonstrating their ability to capture the essential information from the original text dataset, achieving performance comparable to humans, particularly under the BERTScore metric.

Despite their lower performance, extractive models can still be useful, especially when interpretability of results is a critical factor. These models employ simple statistical mechanisms, such as word frequency within the text (SumBasic), KL divergence between distributions (KL-Sum), similarity graphs (LexRank), and latent topics derived via SVD (LSA), thus providing deterministic and verifiable decision rules that clarify why each sentence included in the summary was selected. In an auditing context, where transparency, traceability, and justifiability are essential, such characteristics support auditors in making informed decisions on sensitive matters. Within the class of extractive models, the SumBasic method stands out, ranking as the fourth best overall method according to Table 13, considering its performance across all metrics compared to the others.

Among the methods with the best overall results are the abstractive models NousResearch/Hermes-3-Llama-3.2-3B, Qwen/Qwen2.5-7B-Instruct, and meta-llama/Llama-3.2-3B-Instruct, which consistently outperform across all metrics, for instance, in BERTScore as shown in Table 13. This result suggests that they better capture and preserve the contextual meaning of the original text.

To assess the consistency of each automatic summary across each sample in all iterations, the standard deviation of the metrics was analyzed, revealing the stability of the methods' performance relative to the evaluated metrics. A high standard deviation indicates that the model's performance varies considerably across test runs, while a low standard deviation indicates greater consistency, delivering similar results regardless of the evaluated example or the number of iterations.

For comparative purposes, since all metrics range from zero to one, we classified results into “Low” (*std* < 0.05), “Moderate” (*std*>0.05, and < 0.1), and “High” (*std*>0.1) based on the standard deviation value for each metric. From this perspective, the methods NousResearch/Hermes-3-Llama-3.2-3B, Qwen/Qwen2.5-7B-Instruct, and meta-llama/Llama-3.2-3B-Instruct maintain their prominence with moderate variation, as shown in Table 8.

**Table 8:** Performance consistency classification using standard deviation of results.

**Method**	**ROUGE-1 F1 (Std)**	**ROUGE-2 F1 (Std)**	**ROUGE-L (Std)**	**BLEU (Std)**	**METEOR (Std)**	**BERTScore F1 (Std)**
NousResearch/Hermes-3-Llama-3.2-3B	Moderate	Moderate	Moderate	Moderate	High	Moderate
Qwen/Qwen2.5-0.5B-Instruct	Moderate	Moderate	Moderate	Low	Moderate	Moderate
Qwen/Qwen2.5-1.5B-Instruct	High	High	High	Moderate	High	Moderate
Qwen/Qwen2.5-7B-Instruct	Moderate	Moderate	Moderate	Moderate	Moderate	Moderate
TucanoBR/Tucano-1b1-Instruct	Moderate	Moderate	Moderate	Low	Moderate	Moderate
TucanoBR/Tucano-2b4-Instruct	High	Moderate	Moderate	Low	Moderate	Moderate
Google/gemma-2b-it	High	High	High	Moderate	High	High
Klsum	High	Moderate	Moderate	Low	Moderate	Moderate
Lexrank	High	High	Moderate	Moderate	High	Moderate
Lsa	High	Moderate	Moderate	Low	Moderate	Moderate
Maritaca-ai/sabia-7b	High	Moderate	Moderate	Moderate	High	High
Meta-llama/Llama-3.2-3B-Instruct	Moderate	Moderate	Moderate	Moderate	Moderate	Low
Nicholaskluge/TeenyTinyLlama-460m-Chat	Moderate	Moderate	Low	Low	Moderate	Moderate
Sumbasic	High	High	High	Moderate	High	Moderate
Textrank	Moderate	Moderate	Moderate	Low	Moderate	Moderate

In addition, to verify the consistency of the methods' results, an analysis was conducted to identify and characterize the presence of outliers, considering as such those values greater than three standard deviations, as well as those identified through the Interquartile Range (IQR). Among the 75 model-metric combinations, only 22 exhibited outliers above 1%, indicating a low incidence of outliers and, consequently, supporting the consistency of results across different iterations. Table 9 presents the distribution of outliers by method and metric.

**Table 9:** Percentage of outliers detected by *Z*-score and IQR for different methods and metrics.

**Method**	**Metric**	***N***	**Z_Outliers_%**	**IQR_Outliers_%**
Nicholaskluge/TeenyTinyLlama-460m-Chat	BLEU	14,735	2.586	10.499
TucanoBR/Tucano-1b1-Instruct	BLEU	14,735	2.260	7.112
Klsum	ROUGE-2 F1	14,735	2.138	2.850
Klsum	BLEU	14,735	2.138	6.888
Maritaca-ai/sabia-7b	BLEU	14,735	2.029	12.257
Textrank	BLEU	14,735	1.900	8.789
Maritaca-ai/sabia-7b	ROUGE-2 F1	14,735	1.887	4.269
Nicholaskluge/TeenyTinyLlama-460m-Chat	ROUGE-2 F1	14,735	1.805	4.466
Textrank	METEOR	14,735	1.663	2.613
Klsum	BERTScore F1	14,735	1.663	5.701
Textrank	ROUGE-2 F1	14,735	1.663	1.900
TucanoBR/Tucano-2b4-Instruct	ROUGE-1 F1	14,735	1.636	1.934
Qwen/Qwen2.5-0.5B-Instruct	BLEU	14,735	1.513	3.509
TucanoBR/Tucano-2b4-Instruct	BERTScore F1	147,35	1.513	3.101
Klsum	METEOR	14,735	1.425	3.325
Lsa	ROUGE-2 F1	14,735	1.425	1.425
Lexrank	METEOR	14,735	1.425	3.800
Lexrank	BLEU	14,735	1.425	1.663
Maritaca-ai/sabia-7b	METEOR	14,735	1.391	1.778
TucanoBR/Tucano-2b4-Instruct	BLEU	14,735	1.344	2.246
Qwen/Qwen2.5-1.5B-Instruct	BLEU	14,735	1.289	2.328
Google/gemma-2b-it	BLEU	14,735	1.140	1.608
Nicholaskluge/TeenyTinyLlama-460m-Chat	METEOR	14,735	1.086	5.375

Filtered only values above 1%.

To analyze the reduction of information overload, in addition to quality metrics, textual reduction relative to the original document and its size compared to human performance were assessed. Beyond preserving quality, the best models—NousResearch/Hermes-3-Llama-3.2-3B, Qwen/Qwen2.5-7B-Instruct, and meta-llama/Llama-3.2-3B-Instruct—produced summaries with lengths relatively close to the human average, with mean differences in word count of 10.57%, 3.25%, and 8.13%, respectively, compared to human performance. Table 10 describes the reduction of information overload, reporting the average length of all news articles in words (Orig. Words), the average human summary length across all documents (Ref. Words) and its relative size compared to the average document length (Ref. %), the average length of automatic summaries (Auto Words) and its relative size compared to the average document length (Auto %), and the relative difference between automatic summaries and human performance (Dif %). Supplementary Appendix A.15 compares reference summary samples with automatic summaries generated by the top-performing models.

**Table 10:** Percentage difference in information overload reduction between human summary vs. automatic summary. Sorted by diff. (%).

**Method**	**Type**	**Orig. words**	**Ref. words**	**Auto words**	**Ref. (%)**	**Auto (%)**	**Dif (%)**
Maritaca-ai/sabia-7b	Abstractive	2,726	123	90	4.51	3.30	–26.83
Google/gemma-2b-it	Abstractive	2,726	123	102	4.51	3.74	–17.07
Klsum	Extractive	2,726	123	113	4.51	4.15	–8.13
Nicholaskluge/TeenyTinyLlama-460m-Chat	Abstractive	2,726	123	122	4.51	4.48	–0.81
Sumbasic	Extractive	2,726	123	124	4.51	4.55	0.81
Qwen/Qwen2.5-7B-Instruct	Abstractive	2,726	123	127	4.51	4.66	3.25
TucanoBR/Tucano-1b1-Instruct	Abstractive	2,726	123	128	4.51	4.70	4.07
Qwen/Qwen2.5-1.5B-Instruct	Abstractive	2,726	123	129	4.51	4.73	4.88
TucanoBR/Tucano-2b4-Instruct	Abstractive	2,726	123	129	4.51	4.73	4.88
Qwen/Qwen2.5-0.5B-Instruct	Abstractive	2,726	123	131	4.51	4.81	6.50
Meta-llama/Llama-3.2-3B-Instruct	Abstractive	2,726	123	133	4.51	4.88	8.13
NousResearch/Hermes-3-Llama-3.2-3B	Abstractive	2,726	123	136	4.51	4.99	10.57
Lexrank	Extractive	2,726	123	171	4.51	6.27	39.02
Lsa	Extractive	2,726	123	212	4.51	7.78	72.36
Textrank	Extractive	2,726	123	290	4.51	10.64	135.77

Ordenado por dif (%).

Despite the promising results, it is not possible to draw definitive conclusions without sufficiently conclusive statistical evidence. Thus, to enable comparative assertions, a significance level (α) of 0.05 was established for the entire experiment. Normality tests were then applied.

A normality assessment was carried out using robust methods for large samples (14,735 per method), specifically the Anderson–Darling (AD Test) and Kolmogorov–Smirnov (KS Test), in order to determine the most appropriate hypothesis test to answer the Research Questions (Section 4.4). The AD Test was adopted as the primary metric, while the KS Test was employed as a complementary verification. The results indicated no evidence that any of the datasets follow a normal distribution, as presented in Tables 11–13. For the 14,735 samples, the critical value for the AD statistic was 0.787, leading to rejection across all methods and metrics. Analysis of the *p*-values from the Kolmogorov–Smirnov test further confirmed that none of the result distributions for any metric approximate a normal distribution. Consequently, the use of non-parametric hypothesis testing was required. As the primary approach, the Wilcoxon Signed-Rank Test was employed in a pairwise manner (Method A vs. Method B) to evaluate whether the distribution of values from “Method A” was significantly superior to that of “Method B.”

**Table 11:** Normality test results—Anderson–Darling (AD_statistic).

**Method**	**BERTScore F1**	**BLEU**	**METEOR**	**ROUGE-1 F1**	**ROUGE-2 F1**	**ROUGE-L**
NousResearch/Hermes-3-Llama-3.2-3B	29.77	56.15	42.39	69.31	5.49	29.40
Qwen/Qwen2.5-0.5B-Instruct	3.92	674.17	138.18	6.38	153.81	156.32
Qwen/Qwen2.5-1.5B-Instruct	126.35	296.25	56.14	594.46	42.02	125.39
Qwen/Qwen2.5-7B-Instruct	3.70	89.42	39.92	10.83	19.21	68.15
TucanoBR/Tucano-1b1-Instruct	40.96	1116.53	118.44	9.26	168.26	159.44
TucanoBR/Tucano-2b4-Instruct	235.25	491.28	26.11	112.81	56.36	55.47
Google/gemma-2b-it	657.38	747.67	238.79	486.87	451.69	205.86
Klsum	298.46	1407.12	199.69	47.79	451.36	183.15
Lexrank	12.16	674.93	164.17	9.18	200.53	136.52
Lsa	33.69	670.52	139.81	11.45	230.39	110.26
Maritaca-ai/sabia-7b	112.57	1666.28	244.46	132.77	613.58	126.77
Meta-llama/Llama-3.2-3B-Instruct	9.32	76.45	55.90	23.79	37.85	105.51
Nicholaskluge/TeenyTinyLlama-460m-Chat	107.45	1602.94	119.82	83.45	452.51	157.85
Sumbasic	43.29	83.20	27.09	42.31	10.65	25.12
Textrank	8.70	1179.22	145.10	17.34	236.46	62.60

**Table 12:** Normality test results—Kolmogorov–Smirnov (KS_pvalue).

**Method**	**BERTScore F1**	**BLEU**	**METEOR**	**ROUGE-1 F1**	**ROUGE-2 F1**	**ROUGE-L**
NousResearch/Hermes-3-Llama-3.2-3B	2.98 × 10^−17^	2.19 × 10^−40^	4.11 × 10^−19^	2.37 × 10^−38^	1.66 × 10^−6^	1.44 × 10^−23^
Qwen/Qwen2.5-0.5B-Instruct	2.95 × 10^−2^	3.76 × 10^−307^	5.94 × 10^−56^	5.03 × 10^−4^	5.01 × 10^−53^	1.76 × 10^−73^
Qwen/Qwen2.5-1.5B-Instruct	5.80 × 10^−54^	1.75 × 10^−159^	5.92 × 10^−29^	9.12 × 10^−240^	1.68 × 10^−22^	9.67 × 10^−61^
Qwen/Qwen2.5-7B-Instruct	7.49 × 10^−2^	1.14 × 10^−31^	5.13 × 10^−19^	1.99 × 10^−10^	5.00 × 10^−10^	1.20 × 10^−33^
TucanoBR/Tucano-1b1-Instruct	3.44 × 10^−28^	0	1.24 × 10^−61^	2.34 × 10^−7^	3.54 × 10^−63^	5.36 × 10^−81^
TucanoBR/Tucano-2b4-Instruct	3.59 × 10^−92^	2.09 × 10^−205^	1.33 × 10^−16^	2.34 × 10^−60^	1.06 × 10^−26^	3.25 × 10^−23^
Google/gemma-2b-it	0	0	1.93 × 10^−120^	2.19 × 10^−206^	1.60 × 10^−260^	1.20 × 10^−127^
Klsum	6.99 × 10^−126^	0	2.19 × 10^−121^	6.50 × 10^−29^	1.63 × 10^−265^	7.69 × 10^−82^
Lexrank	6.23 × 10^−16^	4.71 × 10^−314^	1.43 × 10^−78^	1.40 × 10^−10^	4.59 × 10^−95^	9.75 × 10^−126^
Lsa	1.04 × 10^−23^	0	9.79 × 10^−69^	4.76 × 10^−8^	8.79 × 10^−114^	4.51 × 10^−70^
Maritaca-ai/sabia-7b	9.04 × 10^−76^	0	7.66 × 10^−179^	1.02 × 10^−57^	0	2.24 × 10^−81^
Meta-llama/Llama-3.2-3B-Instruct	1.47 × 10^−7^	5.38 × 10^−40^	4.28 × 10^−32^	4.33 × 10^−9^	1.26 × 10^−20^	6.89 × 10^−57^
Nicholaskluge/TeenyTinyLlama-460m-Chat	1.09 × 10^−55^	0	1.14 × 10^−48^	2.22 × 10^−35^	1.07 × 10^−185^	8.33 × 10^−58^
Sumbasic	6.03 × 10^−42^	9.10 × 10^−40^	3.53 × 10^−22^	1.61 × 10^−28^	2.03 × 10^−13^	3.61 × 10^−23^
Textrank	5.96 × 10^−7^	0	9.19 × 10^−109^	1.40 × 10^−12^	7.29 × 10^−107^	1.10 × 10^−36^

**Table 13:** Summary of the Wilcoxon Signed-Rank Test (pairwise).

**Method**	**ROUGE-1 F1**	**ROUGE-2 F1**	**ROUGE-L**	**BLEU**	**METEOR**	**BERTScore F1**	**Score**
NousResearch/Hermes-3-Llama-3.2-3B	12	14	14	14	14	12	80
Qwen/Qwen2.5-7B-Instruct	13	13	13	12	12	14	77
Meta-llama/Llama-3.2-3B-Instruct	12	12	12	13	13	12	74
Sumbasic	11	11	11	11	11	11	66
Qwen/Qwen2.5-1.5B-Instruct	10	10	10	10	10	10	60
Lexrank	6	9	9	9	9	8	50
TucanoBR/Tucano-2b4-Instruct	9	7	6	7	7	9	45
Qwen/Qwen2.5-0.5B-Instruct	8	6	5	6	6	7	38
Google/gemma-2b-it	5	8	6	8	5	4	36
Lsa	4	5	6	5	7	5	32
TucanoBR/Tucano-1b1-Instruct	6	4	4	4	3	6	27
Textrank	1	2	3	2	3	2	13
Nicholaskluge/TeenyTinyLlama-460m-Chat	3	0	1	0	2	3	9
Klsum	2	1	2	1	1	1	8
Maritaca-ai/sabia-7b	0	3	0	2	0	0	5

Sorted by score.

To assess whether one method significantly outperforms another, the Wilcoxon Signed-Rank Test was employed. This is a non-parametric test for paired samples that evaluates whether the median of the differences between two methods (or conditions) is significantly different from zero. Following its application, evidence was obtained regarding the comparative performance across each of the evaluation metrics. Tables B.16, B.17, B.18, B.19 and B.20 in Supplementary Appendix B describes the pairwise results of Wilcox Singed-Rank Test for each evaluation metric. These results are summarized in Table 13, which reports the number of scores (when the p-value is significant) of the baseline method (“Model A”) over the alternative method (“Model B”) in pairwise comparisons for each metric, while the column *Score* represents the cumulative count of scores for the baseline model (“Model A”) over the alternative models (“Model B”).

After identifying the models with the best comparative performance, the next step involved measuring the magnitude of the difference between the medians of the top models in both the most favorable and least favorable scenarios, with the aim of evaluating the extent to which one model stands out relative to the alternative. Table 14 presents the baseline model, the models corresponding to the best- and worst-case scenarios, as well as the respective metrics. Furthermore, Table 14 reports the results obtained when the baseline model outperformed or underperformed for each analyzed metric.

**Table 14:** Magnitude of the difference in medians between the best models in the best- and worst-case scenarios.

**Baseline model**	**Metric**	**Compared model**	**Largest diff**	**Compared model**	**Smallest diff**
NousResearch/Hermes-3-Llama-3.2-3B	BERTScore F1	Maritaca-ai/sabia-7b	0.192399	Sumbasic	0.025466
Meta-llama/Llama-3.2-3B-Instruct	BERTScore F1	Maritaca-ai/sabia-7b	0.191081	Sumbasic	0.024147
Qwen/Qwen2.5-7B-Instruct	BERTScore F1	Maritaca-ai/sabia-7b	0.193555	NousResearch/Hermes-3-Llama-3.2-3B	0.001156
NousResearch/Hermes-3-Llama-3.2-3B	BLEU	Nicholaskluge/TeenyTinyLlama-460m-Chat	0.135554	Qwen/Qwen2.5-7B-Instruct	0.012369
Meta-llama/Llama-3.2-3B-Instruct	BLEU	Nicholaskluge/TeenyTinyLlama-460m-Chat	0.122471	Qwen/Qwen2.5-7B-Instruct	-0.000714
Qwen/Qwen2.5-7B-Instruct	BLEU	Nicholaskluge/TeenyTinyLlama-460m-Chat	0.123185	Sumbasic	0.008521
NousResearch/Hermes-3-Llama-3.2-3B	METEOR	Maritaca-ai/sabia-7b	0.235310	Meta-llama/Llama-3.2-3B-Instruct	0.008221
Meta-llama/Llama-3.2-3B-Instruct	METEOR	Maritaca-ai/sabia-7b	0.227089	Qwen/Qwen2.5-7B-Instruct	0.005301
Qwen/Qwen2.5-7B-Instruct	METEOR	Maritaca-ai/sabia-7b	0.221789	Sumbasic	0.040476
NousResearch/Hermes-3-Llama-3.2-3B	ROUGE-1 F1	Maritaca-ai/sabia-7b	0.281186	Sumbasic	0.045868
Meta-llama/Llama-3.2-3B-Instruct	ROUGE-1 F1	Maritaca-ai/sabia-7b	0.277018	Sumbasic	0.041700
Qwen/Qwen2.5-7B-Instruct	ROUGE-1 F1	Maritaca-ai/sabia-7b	0.277188	Meta-llama/Llama-3.2-3B-Instruct	0.000170
NousResearch/Hermes-3-Llama-3.2-3B	ROUGE-2 F1	Nicholaskluge/TeenyTinyLlama-460m-Chat	0.235764	Qwen/Qwen2.5-7B-Instruct	0.012609
Meta-llama/Llama-3.2-3B-Instruct	ROUGE-2 F1	Nicholaskluge/TeenyTinyLlama-460m-Chat	0.220045	Sumbasic	0.017806
Qwen/Qwen2.5-7B-Instruct	ROUGE-2 F1	Nicholaskluge/TeenyTinyLlama-460m-Chat	0.223155	Meta-llama/Llama-3.2-3B-Instruct	0.003110
NousResearch/Hermes-3-Llama-3.2-3B	ROUGE-L	Maritaca-ai/sabia-7b	0.194257	Qwen/Qwen2.5-7B-Instruct	0.013018
Meta-llama/Llama-3.2-3B-Instruct	ROUGE-L	Maritaca-ai/sabia-7b	0.175403	Sumbasic	0.005116
Qwen/Qwen2.5-7B-Instruct	ROUGE-L	Maritaca-ai/sabia-7b	0.181239	Meta-llama/Llama-3.2-3B-Instruct	0.005836

The models NousResearch/Hermes-3-Llama-3.2-3B and Qwen/Qwen2.5-7B-Instruct were pre-trained using English-language datasets, whereas meta-llama/Llama-3.2-3B-Instruct was trained on multilingual corpora (Table 4). Despite being trained entirely or predominantly in foreign languages, these models achieved the best performance across all summary quality evaluation criteria and pairwise comparisons. For the BERTScore metric, the difference was 0.19 or 19%, meaning that compared to human performance, the three top-performing models were 19% superior to the compared model. Conversely, the models with the weakest relative results compared to the top three were those pre-trained in Portuguese, namely maritaca-ai/sabia-7b and nicholasKluge/TeenyTinyLlama-460m-Chat. Meanwhile, the models that exhibited the smallest performance differences were the extractive sumbasic and the abstractive NousResearch/Hermes-3-Llama-3.2-3B and Qwen/Qwen2.5-7B-Instruct.

The results of all evaluations indicate, from the perspective of an auditor, that these models are technically reliable, as they demonstrate consistency in performance, highlight the most relevant information, and maintain an average summary length comparable to human performance. Thus, by leveraging automated methods to contribute to reducing informational overload, these models can support the auditing process in the analytical phase, enhancing efficiency and effectiveness in information gathering and preparing teams for the operational phase in less time.

### 6.2 Threats to validity

For the evaluation of the experiment, it is necessary to consider factors that may influence the results, characterized as threats to internal and external validity.

- Internal validity: the process of classifying the news articles was conducted by two annotators. As this is a manual and intensive activity, there is a possibility of classification errors. To mitigate this risk, five evaluators intervened in cases of disagreement between annotators regarding the categorization of the news.
- External validity: the number of methods trained exclusively in Brazilian Portuguese or multilingual corpora is very limited compared to those developed in English. Models trained in the same language as the dataset may achieve superior or more consistent performance relative to multilingual or English-adapted models. To mitigate this threat, comparisons were balanced by prioritizing models trained or fine-tuned in Brazilian Portuguese.

## 7 Conclusion and future work

The auditing process is generally characterized as costly, time-consuming, and resource-intensive, requiring substantial human and material effort. In this context, it becomes necessary to implement solutions and techniques that enable the automation of the analysis of corruption allegations. This process is typically divided into two stages: in the first, elements and evidence of corruption—such as suppliers, contracts, employees, clients, and other stakeholders—are identified, assessing the plausibility and consistency of the allegations and signs of fraud; in the second stage, the investigation itself is carried out.

For building the knowledge required in auditing activities, it is essential to collect information directly related to the audit's objectives. In this phase, various sources are consulted, including websites. To support the information-gathering process, webscraping techniques can be applied to extract large-scale data from health-related websites. Furthermore, to assist in analyzing this substantial volume of data, NLP techniques, such as text summarization, can be employed, significantly reducing the time and resources required for the analysis and collection of evidence of potential irregularities.

In this context, aiming to support, improve, and optimize the collection of relevant information that may assist in combating irregularities, this study presents the results of applying 15 automatic text summarization methods to a set of health-related news articles with indications of irregularities. The objective was to evaluate whether such methods can contribute to the auditing process by reducing informational overload, as well as to identify which are most effective for this task.

In this controlled experiment, using a curated dataset of 421 news samples, automatic summaries were generated through 15 methods, each repeated over 35 rounds. The results were robustly evaluated based on multiple performance metrics [ROUGE-N (Lin, 2004), ROUGE-L (Lin, 2004), BLEU (Papineni et al., 2002), METEOR (Lavie and Agarwal, 2007), and BERTScore (Zhang et al. 2020)]. In addition to evaluating summary quality across multiple metrics, the analysis included consistency through standard deviation, the presence of outliers, and the degree of informational overload reduction. Moreover, all methods were subjected to the Wilcoxon Signed-Rank Test (paired), the results of which are presented in this study.

Among the methods with the best performance in terms of summary quality and comparative results, the models NousResearch/Hermes-3-Llama-3.2-3B, Qwen/Qwen2.5-7B-Instruct, and meta-llama/Llama-3.2-3B-Instruct stand out. These models consistently outperformed others across the various evaluation metrics, demonstrating a superior ability to capture and preserve the contextual meaning of the original text while adequately synthesizing key information, when compared to human performance.

From an auditor's perspective, these models prove to be technically reliable, as they provide consistent results, highlight the most relevant information, and maintain an average summary length comparable to human performance. Therefore, by leveraging automated methods to reduce informational overload, these models can support the auditing process in the analytical phase, increasing efficiency and effectiveness in information gathering and enabling teams to prepare for the operational phase in less time.

For future work, although these models require relatively fewer computational resources compared to larger models, their implementation and execution still demand specialized knowledge and significant resources. In this regard, there is room for exploring complexity reduction techniques, such as quantization methods, which may enable more efficient use of these models in practical scenarios with limited resources. Finally, to further optimize the reduction of informational overload, it would be possible to summarize groups of texts rather than individual texts, following the application of topic modeling methods.

## Data Availability

The raw data supporting the conclusions of this article will be made available by the authors, without undue reservation.

## Footnotes

1
https://jupyter.org/

2
https://docs.pola.rs/

3
https://www.python.org/

4
https://colab.google/

5
https://www.python.org/
