# Supplementary material for: Small language models applied in text summarization task of health-related news to improve public health audit: an experimental case study
Source: Front Artif Intell. 2026 Feb 5;9:1708993. doi: 10.3389/frai.2026.1708993 (PMC12916652; doi:10.3389/frai.2026.1708993)
Supplement: Supplementary file 1 [file Data_Sheet_1.pdf]

Appendix A. Appendix - Amostras

Table A.15: Comparison between human and automatic summaries.

**Human Summary 1**
O Conselho Regional de Medicina do Estado de Goiás (Cremego) informou que, até o momento, não foi notificado sobre qualquer ação da Polícia Federal referente às investigações sobre a conduta do médico oftalmologista Farley Vinícius Alcântara, por ele atestar que Gabriela Santiago Ribeiro Cid, esposa do ex-ajudante de ordens de Jair Bolsonaro, Mauro Cid, foi imunizada contra a Covid-19 no município. Segundo as investigações da operação venire, foi relatado que dados da vacina foram falsificados, entre eles, a do ex-presidente Jair Bolsonaro; de sua filha mais nova, Laura Bolsonaro; de Mauro Cid, um dos auxiliares de Bolsonaro presos; da esposa de Cid; e da filha de Cid. Seis pessoas já foram presas e outros 16 mandados foram cumpridos.
**Automatic Summaries**
**NousResearch/Hermes-3-Llama-3.2-3B:** O Conselho Regional de Medicina do Estado de Goiás (Cremego) informou que não foi notificado sobre ações da Polícia Federal relacionadas a investigações contra o médico Farley Vinícius Alcântara. O oftalmologista é suspeito de ter fornecido um documento atestando que a mulher do ex-ajudante de ordens de Jair Bolsonaro, Mauro Cid, se imunizou contra a Covid-19 no município de Cabeceiras. A Polícia Federal deflagrou a Operação Venire, apurando que dados de vacina contra a Covid foram falsificados, envolvendo ex-presidente Jair Bolsonaro, sua filha Laura, Mauro Cid, esposa de Cid e filha de Cid. Foram presos seis pessoas e cumpridos 16 mandados. Farley, servidor da Secretaria de Saúde do Distrito Federal, copiou dados de vacinação de uma enfermeira de Cabeceiras para falsificar um cartão de imunização
**Qwen/Qwen2.5-7B-Instruct:** A Polícia Federal deflagrou a Operação Venire em 3 de maio, investigando fraudes em registros de vacinação contra a COVID-19 envolvendo seis pessoas, incluindo membros da família Bolsonaro e assessores próximos. Farley Vinícius Alcântara, médico oftalmologista, foi acusado de falsificar dados de vacinação para a esposa de Mauro Cid. O crime não foi consumado devido à falta de distribuição de lotes de vacinas para o Rio de Janeiro. Farley é servidor da Secretaria de Saúde do DF, mas trabalha em uma clínica no Rio de Janeiro. O prefeito de Cabeceiras negou que Bolsonaro ou seus assessores se vacinaram no município. O caso está sendo investigado e pode resultar em processos administrativos contra o médico.
**meta-llama/Llama-3.2-3B-Instruct:** Aqui está um resumo objetivo e conciso do conteúdo em português: A Polícia Federal (PF) deflagrou a Operação Venire, que envolveu a investigação de um esquema de fraude em registros de vacinação contra a Covid-19. O médico Farley Vinícius Alcântara, que trabalha em uma clínica particular de olhos no Rio de Janeiro, foi acusado de copiar dados de vacinação de uma enfermeira de Cabeceiras, interior de Goiás, para falsificar um cartão de imunização para a esposa de Mauro Cid, um dos auxiliares de Jair Bolsonaro presos. O prefeito de Cabeceiras, Everton Francisco de Matos (PDT), conhecido como Tuta, afirmou que nenhum membro da família de Bolsonaro ou qualquer assessor se vacinou no município. Ele também afirmou que o município está isento disso e que a atitude do médico Farley está prejudicando
**Human Summary 2**
O ministro da Controladoria-Geral da União, Wagner Rosário, afirmou à CNN que a CGU abriu investigação sobre o caso Davati. A apuração busca esclarecer o encontro entre o empresário Luiz Paulo Dominguetti e o ex-diretor do Ministério da Saúde, Roberto Dias, onde teria havido pedido de propina de US$ 1 por dose de vacina. Segundo Rosário, o sistema do Ministério registrou proposta de 300 milhões de doses pela Davati, mas sem vínculo oficial com Dominguetti. Ele também confirmou a entrada do empresário no ministério em março de 2021. O ministro disse que, até o momento, não há irregularidades no contrato da vacina Covaxin, mas apontou que houve três problemas no invoice, um tipo de nota fiscal, mas que todos já foram corrigidos. Rosário declarou estar pronto para depor na CPI da Pandemia, se convocado, e evitou comentar as críticas do presidente Jair Bolsonaro à comissão.
**Automatic Summaries**

NousResearch/Hermes-3-Llama-3.2-3B: Em entrevista exclusiva à CNN, o ministro-chefe da Controladoria-Geral da União, Wagner Rosário, afirmou que a CGU abriu uma investigação para apurar o caso Davati e uma suposta cobrança de propina. A CGU quer entender melhor o que aconteceu no encontro entre o empresário Luiz Paulo Dominguetti Pereira e o então diretor do Ministério da Saúde Roberto Ferreira Dias, em um restaurante de um shopping de Brasília. A CGU também apurou que Dominguetti esteve no prédio do Ministério da Saúde no dia 26 de março de 2021, com entrada às 13h39. Rosário, porém, não soube informar com quem o empresário esteve. Wagner Rosário disse que "processualmente" não há irregularidades nos contratos de contratação e importação da vacina indiana Covaxin, mas o relatório ainda não está concluído. O departamento do Ministério da Saúde que cuida dessa área identificou três

Qwen/Qwen2.5-7B-Instruct: Aqui está uma versão concisa e objetiva do texto em português: O ministro-chefe da Controladoria-Geral da União, Wagner Rosário, concedeu uma entrevista à CNN sobre investigações relacionadas à pandemia de COVID-19 no Brasil. Ele afirmou que a CGU está investigando um suposto pedido de propina e um encontro entre um empresário e um ex-dirigente do Ministério da Saúde. Rosário também declarou que não há irregularidades processuais nas contratações da vacina Covaxin, apesar de problemas encontrados no processo de importação das vacinas. Ele indicou que esses problemas foram corrigidos antes da conclusão do contrato. O ministro admitiu a possibilidade de depor à CPI da Pandemia, caso seja convocado pelos senadores. Ele também comentou sobre críticas feitas pelo presidente Jair Bolsonaro à CPI, afirmando que cada cidadão deve avaliar o comportamento dos membros da comissão. Resumindo, a principal informação é que a CGU está investig

meta-llama/Llama-3.2-3B-Instruct: Aqui está um resumo objetivo e conciso do conteúdo em português: O ministro-chefe da Controladoria-Geral da União, Wagner Rosário, afirmou que a CGU abriu uma investigação para apurar o caso Davati e uma suposta cobrança de propina. Ele também admitiu que está pronto para depor à CPI da Pandemia, caso seja acionado pelos senadores que integram a comissão. Rosário ressaltou que o relatório ainda não está concluído e, por isso, não poderia se manifestar definitivamente sobre o caso. Ele também afirmou que não há irregularidades nos contratos de contratação e importação da vacina indiana Covaxin. A CGU apurou que o empresário Luiz Paulo Dominguetti Pereira teria recebido um pedido de propina de US$ 1 por dose de vacina da Astrazeneca em troca de fechar contrato com o Ministério da Saúde. No entanto, Rosário não soube informar com quem o empresário esteve

Appendix B. Appendix - Pairwise Wilcox Singed-Rank Test

Table B.16: Pairwise p-value for ROUGE-1 F1 scores among evaluated models.

Model	Hermes-3-Llama-3.2-3B	Qwen2.5-0.5B	Qwen2.5-1.5B	Qwen2.5-7B	Tucano-1b1	Tucano-2b4	gemma-2b-it	klsum	lexrank	lsa	sabia-7b	Llama-3.2-3B	TeenyTinyLlama	sumbasic	textrank
Hermes-3-Llama-3.2-3B	NaN	0.0	0.0	0.792063	0.0	0.0	0.0	0.0	0.0	0.0	0.0	0.066455	0.0	0.0	0.0
Qwen2.5-0.5B	1.0	NaN	1.0	1.0	0.0	1.0	0.0	0.0	0.0	0.0	0.0	1.0	0.0	1.0	0.0
Qwen2.5-1.5B	1.0	0.0	NaN	1.0	0.0	0.0	0.0	0.0	0.0	0.0	0.0	1.0	0.0	1.0	0.0
Qwen2.5-7B	0.207937	0.0	0.0	NaN	0.0	0.0	0.0	0.0	0.0	0.0	0.0	0.009585	0.0	0.0	0.0
Tucano-1b1	1.0	1.0	1.0	1.0	NaN	1.0	0.0	0.0	0.817954	0.0	0.0	1.0	0.0	1.0	0.0
Tucano-2b4	1.0	0.0	1.0	1.0	0.0	NaN	0.0	0.0	0.0	0.0	0.0	1.0	0.0	1.0	0.0
gemma-2b-it	1.0	1.0	1.0	1.0	1.0	1.0	NaN	0.0	1.0	0.0	0.0	1.0	0.0	1.0	0.0
klsum	1.0	1.0	1.0	1.0	1.0	1.0	1.0	NaN	1.0	1.0	0.0	1.0	1.0	1.0	0.0
lexrank	1.0	1.0	1.0	1.0	0.182046	1.0	0.0	0.0	NaN	0.0	0.0	1.0	0.0	1.0	0.0
lsa	1.0	1.0	1.0	1.0	1.0	1.0	1.0	0.0	1.0	NaN	0.0	1.0	0.0	1.0	0.0
sabia-7b	1.0	1.0	1.0	1.0	1.0	1.0	1.0	1.0	1.0	1.0	NaN	1.0	1.0	1.0	0.999998
Llama-3.2-3B	0.933545	0.0	0.0	0.990415	0.0	0.0	0.0	0.0	0.0	0.0	0.0	NaN	0.0	0.0	0.0
TeenyTinyLlama	1.0	1.0	1.0	1.0	1.0	1.0	1.0	0.0	1.0	1.0	0.0	1.0	NaN	1.0	0.0
sumbasic	1.0	0.0	0.0	1.0	0.0	0.0	0.0	0.0	0.0	0.0	0.0	1.0	0.0	NaN	0.0
textrank	1.0	1.0	1.0	1.0	1.0	1.0	1.0	1.0	1.0	1.0	0.000002	1.0	1.0	1.0	NaN

Table B.17: Pairwise p-value for ROUGE-2 F1 scores among evaluated models.

Model	Hermes-3-Llama-3.2-3B	Qwen2.5-0.5B	Qwen2.5-1.5B	Qwen2.5-7B	Tucano-1b1	Tucano-2b4	gemma-2b-it	klsum	lexrank	lsa	sabia-7b	Llama-3.2-3B	TeenyTinyLlama	sumbasic	textrank
Hermes-3-Llama-3.2-3B	NaN	0.0	0.0	0.0	0.0	0.0	0.0	0.0	0.0	0.0	0.0	0.0	0.0	0.0	0.0
Qwen2.5-0.5B	1.0	NaN	1.0	1.0	0.0	1.0	1.0	0.0	1.0	0.0	0.0	1.0	0.0	1.0	0.0
Qwen2.5-1.5B	1.0	0.0	NaN	1.0	0.0	0.0	0.0	0.0	0.0	0.0	0.0	1.0	0.0	1.0	0.0
Qwen2.5-7B	1.0	0.0	0.0	NaN	0.0	0.0	0.0	0.0	0.0	0.0	0.0	0.007489	0.0	0.0	0.0
Tucano-1b1	1.0	1.0	1.0	1.0	NaN	1.0	1.0	0.0	1.0	1.0	0.0	1.0	0.0	1.0	0.0
Tucano-2b4	1.0	0.0	1.0	1.0	0.0	NaN	0.981377	0.0	1.0	0.0	0.0	1.0	0.0	1.0	0.0
gemma-2b-it	1.0	0.0	1.0	1.0	0.0	0.018623	NaN	0.0	1.0	0.0	0.0	1.0	0.0	1.0	0.0
klsum	1.0	1.0	1.0	1.0	1.0	1.0	1.0	NaN	1.0	1.0	1.0	1.0	0.0	1.0	0.999992
lexrank	1.0	0.0	1.0	1.0	0.0	0.0	0.0	0.0	NaN	0.0	0.0	1.0	0.0	1.0	0.0
lsa	1.0	1.0	1.0	1.0	0.0	1.0	1.0	0.0	1.0	NaN	0.0	1.0	0.0	1.0	0.0
sabia-7b	1.0	1.0	1.0	1.0	1.0	1.0	1.0	0.0	1.0	1.0	NaN	1.0	0.0	1.0	0.000004
Llama-3.2-3B	1.0	0.0	0.0	0.992511	0.0	0.0	0.0	0.0	0.0	0.0	0.0	NaN	0.0	0.0	0.0
TeenyTinyLlama	1.0	1.0	1.0	1.0	1.0	1.0	1.0	1.0	1.0	1.0	1.0	1.0	NaN	1.0	1.0
sumbasic	1.0	0.0	0.0	1.0	0.0	0.0	0.0	0.0	0.0	0.0	0.0	1.0	0.0	NaN	0.0
textrank	1.0	1.0	1.0	1.0	1.0	1.0	1.0	0.000008	1.0	1.0	0.999996	1.0	0.0	1.0	NaN

Table B.18: Pairwise p-value for ROUGE-L scores among evaluated models.

Model	Hermes-3-Llama-3.2-3B	Qwen2.5-0.5B	Qwen2.5-1.5B	Qwen2.5-7B	Tucano-1b1	Tucano-2b4	gemma-2b-it	klsum	lexrank	lsa	sabia-7b	Llama-3.2-3B	TeenyTinyLlama	sumbasic	textrank
NousResearch/Hermes-3-Llama-3.2-3B	NaN	0.0	0.0	0.0	0.0	0.0	0.0	0.0	0.0	0.0	0.0	0.0	0.0	0.0	0.0
Qwen/Qwen2.5-0.5B-Instruct	1.0	NaN	1.0	1.0	0.0	1.0	0.999955	0.0	1.0	1.0	0.0	1.0	0.0	1.0	0.0
Qwen/Qwen2.5-1.5B-Instruct	1.0	0.0	NaN	1.0	0.0	0.0	0.0	0.0	0.0	0.0	0.0	1.0	0.0	1.0	0.0
Qwen/Qwen2.5-7B-Instruct	1.0	0.0	0.0	NaN	0.0	0.0	0.0	0.0	0.0	0.0	0.0	0.0	0.0	0.0	0.0
TucanoBR/Tucano-1b1-Instruct	1.0	1.0	1.0	1.0	NaN	1.0	1.0	0.0	1.0	1.0	0.0	1.0	0.0	1.0	0.0
TucanoBR/Tucano-2b4-Instruct	1.0	0.0	1.0	1.0	0.0	NaN	0.115308	0.0	1.0	0.16874	0.0	1.0	0.0	1.0	0.0
google/gemma-2b-it	1.0	0.000045	1.0	1.0	0.0	0.884692	NaN	0.0	1.0	0.92483	0.0	1.0	0.0	1.0	0.0
klsum	1.0	1.0	1.0	1.0	1.0	1.0	1.0	NaN	1.0	1.0	0.0	1.0	0.0	1.0	0.985231
lexrank	1.0	0.0	1.0	1.0	0.0	0.0	0.0	0.0	NaN	0.0	0.0	1.0	0.0	1.0	0.0
lsa	1.0	0.0	1.0	1.0	0.0	0.83126	0.07517	0.0	1.0	NaN	0.0	1.0	0.0	1.0	0.0
maritaca-ai/sabia-7b	1.0	1.0	1.0	1.0	1.0	1.0	1.0	1.0	1.0	1.0	NaN	1.0	1.0	1.0	1.0
meta-llama/Llama-3.2-3B-Instruct	1.0	0.0	0.0	1.0	0.0	0.0	0.0	0.0	0.0	0.0	0.0	NaN	0.0	0.0	0.0
nicholasKluge/TeenyTinyLlama-460m-Chat	1.0	1.0	1.0	1.0	1.0	1.0	1.0	1.0	1.0	1.0	0.0	1.0	NaN	1.0	1.0
sumbasic	1.0	0.0	0.0	1.0	0.0	0.0	0.0	0.0	0.0	0.0	0.0	1.0	0.0	NaN	0.0
textrank	1.0	1.0	1.0	1.0	1.0	1.0	1.0	0.014769	1.0	1.0	0.0	1.0	0.0	1.0	NaN

Table B.19: Pairwise p-value for BLEU scores among evaluated models.

Model	Hermes-3-Llama-3.2-3B	Qwen2.5-0.5B	Qwen2.5-1.5B	Qwen2.5-7B	Tucano-1b1	Tucano-2b4	gemma-2b-it	klsum	lexrank	lsa	sabia-7b	Llama-3.2-3B	TeenyTinyLlama	sumbasic	textrank
Hermes-3-Llama-3.2-3B	NaN	0.0	0.0	0.0	0.0	0.0	0.0	0.0	0.0	0.0	0.0	0.0	0.0	0.0	0.0
Qwen2.5-0.5B-Instruct	1.0	NaN	1.0	1.0	0.0	0.999995	1.0	0.0	1.0	0.000001	0.0	1.0	0.0	1.0	0.0
Qwen2.5-1.5B-Instruct	1.0	0.0	NaN	1.0	0.0	0.0	0.0	0.0	0.0	0.0	0.0	1.0	0.0	1.0	0.0
Qwen2.5-7B-Instruct	1.0	0.0	0.0	NaN	0.0	0.0	0.0	0.0	0.0	0.0	0.0	0.995113	0.0	0.0	0.0
Tucano-1b1-Instruct	1.0	1.0	1.0	1.0	NaN	1.0	1.0	0.0	1.0	1.0	0.0	1.0	0.0	1.0	0.0
Tucano-2b4-Instruct	1.0	0.000005	1.0	1.0	0.0	NaN	1.0	0.0	1.0	0.0	0.0	1.0	0.0	1.0	0.0
gemma-2b-it	1.0	0.0	1.0	1.0	0.0	0.0	NaN	0.0	0.999549	0.0	0.0	1.0	0.0	1.0	0.0
klsum	1.0	1.0	1.0	1.0	1.0	1.0	1.0	NaN	1.0	1.0	0.999847	1.0	0.0	1.0	0.999294
lexrank	1.0	0.0	1.0	1.0	0.0	0.0	0.000451	0.0	NaN	0.0	0.0	1.0	0.0	1.0	0.0
lsa	1.0	0.999999	1.0	1.0	0.0	1.0	1.0	0.0	1.0	NaN	0.0	1.0	0.0	1.0	0.0
sabia-7b	1.0	1.0	1.0	1.0	1.0	1.0	1.0	0.000153	1.0	1.0	NaN	1.0	0.0	1.0	0.1481
Llama-3.2-3B-Instruct	1.0	0.0	0.0	0.004887	0.0	0.0	0.0	0.0	0.0	0.0	0.0	NaN	0.0	0.0	0.0
TeenyTinyLlama-460m-Chat	1.0	1.0	1.0	1.0	1.0	1.0	1.0	1.0	1.0	1.0	1.0	1.0	NaN	1.0	1.0
sumbasic	1.0	0.0	0.0	1.0	0.0	0.0	0.0	0.0	0.0	0.0	0.0	1.0	0.0	NaN	0.0
textrank	1.0	1.0	1.0	1.0	1.0	1.0	1.0	0.000706	1.0	1.0	0.8519	1.0	0.0	1.0	NaN

Table B.20: Pairwise p-value for METEOR scores among evaluated models.

Model	Hermes-3-Llama-3.2-3B	Qwen2.5-0.5B	Qwen2.5-1.5B	Qwen2.5-7B	Tucano-1b1	Tucano-2b4	gemma-2b-it	klsum	lexrank	lsa	sabia-7b	Llama-3.2-3B	TeenyTinyLlama	sumbasic	textrank
Hermes-3-Llama-3.2-3B	NaN	0.0	0.0	0.0	0.0	0.0	0.0	0.0	0.0	0.0	0.0	0.000462	0.0	0.0	0.0
Qwen2.5-0.5B	1.0	NaN	1.0	1.0	0.0	1.0	0.013323	0.0	1.0	1.0	0.0	1.0	0.0	1.0	0.0
Qwen2.5-1.5B	1.0	0.0	NaN	1.0	0.0	0.0	0.0	0.0	0.0	0.0	0.0	1.0	0.0	1.0	0.0
Qwen2.5-7B	1.0	0.0	0.0	NaN	0.0	0.0	0.0	0.0	0.0	0.0	0.0	1.0	0.0	0.0	0.0
Tucano-1b1	1.0	1.0	1.0	1.0	NaN	1.0	1.0	0.0	1.0	1.0	0.0	1.0	0.0	1.0	0.06444
Tucano-2b4	1.0	0.0	1.0	1.0	0.0	NaN	0.0	0.0	1.0	0.862053	0.0	1.0	0.0	1.0	0.0
gemma-2b-it	1.0	0.986677	1.0	1.0	0.0	1.0	NaN	0.0	1.0	1.0	0.0	1.0	0.0	1.0	0.0
klsum	1.0	1.0	1.0	1.0	1.0	1.0	1.0	NaN	1.0	1.0	0.0	1.0	1.0	1.0	1.0
lexrank	1.0	0.0	1.0	1.0	0.0	0.0	0.0	0.0	NaN	0.0	0.0	1.0	0.0	1.0	0.0
lsa	1.0	0.0	1.0	1.0	0.0	0.137947	0.0	0.0	1.0	NaN	0.0	1.0	0.0	1.0	0.0
sabia-7b	1.0	1.0	1.0	1.0	1.0	1.0	1.0	1.0	1.0	1.0	NaN	1.0	1.0	1.0	1.0
Llama-3.2-3B	0.999538	0.0	0.0	0.0	0.0	0.0	0.0	0.0	0.0	0.0	0.0	NaN	0.0	0.0	0.0
TeenyTinyLlama	1.0	1.0	1.0	1.0	1.0	1.0	1.0	0.0	1.0	1.0	0.0	1.0	NaN	1.0	1.0
sumbasic	1.0	0.0	0.0	1.0	0.0	0.0	0.0	0.0	0.0	0.0	0.0	1.0	0.0	NaN	0.0
textrank	1.0	1.0	1.0	1.0	0.93556	1.0	1.0	0.0	1.0	1.0	0.0	1.0	0.0	1.0	NaN

Table B.21: Pairwise p-value for BERTScore F1 scores among evaluated models.

Model	Hermes-3-Llama-3.2-3B	Qwen2.5-0.5B	Qwen2.5-1.5B	Qwen2.5-7B	Tucano-1b1	Tucano-2b4	gemma-2b-it	klsum	lexrank	lsa	sabia-7b	Llama-3.2-3B	TeenyTinyLlama	sumbasic	textrank
Hermes-3-Llama-3.2-3B	NaN	0.0	0.0	1.0	0.0	0.0	0.0	0.0	0.0	0.0	0.0	0.449384	0.0	0.0	0.0
Qwen2.5-0.5B	1.0	NaN	1.0	1.0	0.0	1.0	0.0	0.0	1.0	0.0	0.0	1.0	0.0	1.0	0.0
Qwen2.5-1.5B	1.0	0.0	NaN	1.0	0.0	0.0	0.0	0.0	0.0	0.0	0.0	1.0	0.0	1.0	0.0
Qwen2.5-7B	0.0	0.0	0.0	NaN	0.0	0.0	0.0	0.0	0.0	0.0	0.0	0.0	0.0	0.0	0.0
Tucano-1b1	1.0	1.0	1.0	1.0	NaN	1.0	0.0	0.0	1.0	0.0	0.0	1.0	0.0	1.0	0.0
Tucano-2b4	1.0	0.0	1.0	1.0	0.0	NaN	0.0	0.0	0.0	0.0	0.0	1.0	0.0	1.0	0.0
gemma-2b-it	1.0	1.0	1.0	1.0	1.0	1.0	NaN	0.0	1.0	1.0	0.0	1.0	0.024403	1.0	0.000063
klsum	1.0	1.0	1.0	1.0	1.0	1.0	1.0	NaN	1.0	1.0	0.0	1.0	1.0	1.0	1.0
lexrank	1.0	0.0	1.0	1.0	0.0	1.0	0.0	0.0	NaN	0.0	0.0	1.0	0.0	1.0	0.0
lsa	1.0	1.0	1.0	1.0	1.0	1.0	0.0	0.0	1.0	NaN	0.0	1.0	0.0	1.0	0.0
sabia-7b	1.0	1.0	1.0	1.0	1.0	1.0	1.0	1.0	1.0	1.0	NaN	1.0	1.0	1.0	1.0
Llama-3.2-3B	0.550616	0.0	0.0	1.0	0.0	0.0	0.0	0.0	0.0	0.0	0.0	NaN	0.0	0.0	0.0
TeenyTinyLlama	1.0	1.0	1.0	1.0	1.0	1.0	0.975597	0.0	1.0	1.0	0.0	1.0	NaN	1.0	0.0
sumbasic	1.0	0.0	0.0	1.0	0.0	0.0	0.0	0.0	0.0	0.0	0.0	1.0	0.0	NaN	0.0
textrank	1.0	1.0	1.0	1.0	1.0	1.0	0.999937	0.0	1.0	1.0	0.0	1.0	1.0	1.0	NaN
